# Characterization of radioresistant epithelial stem cell heterogeneity in the damaged mouse intestine

**DOI:** 10.1038/s41598-020-64987-1

**Published:** 2020-05-22

**Authors:** Taku Sato, Miwako Sase, Shun Ishikawa, Mihoko Kajita, Jumpei Asano, Toshiro Sato, Yoshiyuki Mori, Toshiaki Ohteki

**Affiliations:** 1 Department of Biodefense Research, Medical Research Institute, Tokyo Medical and Dental University (TMDU), Tokyo, 113-8510 Japan; 2 PRESTO, Japan Science and Technology Agency, Saitama, 332-0012 Japan; 3 Department of Dentistry, Oral and Maxillofacial Surgery, Jichi Medical University, Tochigi, 329-0498 Japan; 4 Department of Gastroenterology, Keio University School of Medicine, Tokyo, 160-8582 Japan

**Keywords:** Intestinal stem cells, Regeneration

## Abstract

The small intestine has a robust regenerative capacity, and various cell types serve as “cells-of-origin” in the epithelial regeneration process after injury. However, how much each population contributes to regeneration remains unclear. Using lineage tracing, we found that Lgr5-expressing cell derivatives contained radioresistant intestinal stem cells (ISCs) crucial for epithelial regeneration in the damaged intestine after irradiation. Single-cell qRT-PCR analysis showed that surviving Lgr5-expressing cell derivatives in the damaged intestine are remarkably heterogeneous, and that the expression levels of a YAP-target gene *Sca1* were inversely correlated with their “stemness”, suggesting that the YAP/Wnt signal balance in surviving crypt epithelial cells determines the cellular contribution to epithelial regeneration. Single-cell RNA sequencing of Sca1^–^Lgr5-derivatives revealed that expression of a tetraspanin family member CD81 correlated well with the expression of ISC- and proliferation-related genes. Consistent with these findings, organoid-forming ability was confined to the CD81^hi^Sca1^–^ fraction within the damaged crypt epithelial cells. Characterization of radioresistant epithelial stem cell heterogeneity in the damaged intestine may contribute to therapeutic strategies for gastrointestinal diseases.

## Introduction

Intestinal stem cells (ISCs) continuously produce epithelial cells to preserve the function of the intestinal epithelium both in the steady-state and after tissue injury^1^. Although total body irradiation (TBI) causes apoptosis in the proliferating crypt epithelial cells, including transit-amplifying (TA) cells and ISCs, surviving radio-resistant cells proliferate massively to replenish the lost ISC pool and ISC-derived epithelial cells. The “cell-of-origin” for this epithelial regeneration after injury has been investigated by genetic lineage tracing studies. The “+4” ISCs, located at the +4 position from the crypt bottom, are quiescent^2^ and express specific genes such as *Bmi1*^3^, *mTert*^4^, *Lrig1*^5^, and *Hopx*^6^. Genetic lineage tracing of these markers showed that the quiescent ISCs awaken upon crypt damage and start to proliferate to restore the ISC pool, at least in part. As an alternative mechanism, committed progenitors “dedifferentiate” into ISCs upon damage. Secretory progenitors, which express *Dll1*, encoding a Notch surface receptor ligand, give rise to Paneth cells, goblet cells, endocrine cells, and tuft cells in the steady state, but reacquire ISC potential after irradiation injury^7^. Furthermore, differentiated secretory epithelial cells, including enteroendocrine cells^8^ and Paneth cells^9,10^, also regain stem cell properties during intestinal damage. A population within the Lgr5-expressing ISCs was identified as quiescent label retaining cells (LRCs)^11^. In this context, a fraction of Lgr5^hi^ ISCs expressing Mex3a is a candidate for the radio-resistant quiescent ISCs including LRCs^12^. Consistently, the depletion of Lgr5^+^ cells *in vivo* causes defective epithelial regeneration after irradiation^13^. In another aspect, YAP signal activation in the intestinal epithelium is essential for damage induced regeneration after irradiation exposure^14,15^, parasite infection^16^, and chemically-induced colitis^17^.

Although a variety of cells are synchronously involved in the damage-induced epithelial regeneration, it remains unclear whether or not they overlap each other and to what degree each population contributes to the overall epithelial regeneration. Here, using a combination of genetic lineage tracing, single-cell gene expression profiling, and organoid-formation assays, we characterized the heterogeneity of epithelial stem cells in the irradiation-damaged intestine. Finally, in genetically unmodified mice, we confirmed that the CD81^hi^Sca1^−^ cell fraction in the damaged intestine is the important source for regeneration.

## Results

### Lgr5^hi^ cells contain the cellular origin for irradiation-induced epithelial regeneration

**Figure 1:**
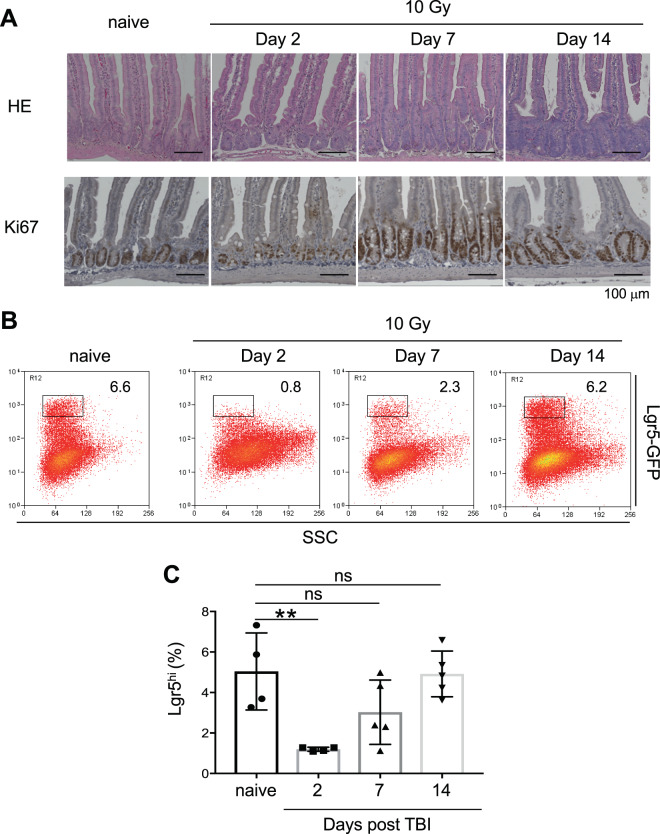
Lgr5-GFP^hi^ Cells Disappear from the Crypt after Irradiation Exposure. (**A**) Representative H&E- and Ki67-stained sections of WT mouse jejunum before and on the indicated days after 10 Gy irradiation (n = 3 for each time point). Scale bars, 100 µm. (**B**,**C**) Representative FACS profiles of Lgr5-GFP expression (**B**) and the frequency of Lgr5^hi^ ISCs (**C**) in crypt epithelial cells before and on the indicated days after 10 Gy irradiation in *Lgr5*^*ki*^ mice (n = 4-5 for each time point). Plotted cells in B were gated on live EpCAM^hi^ cells. Data are means with SD. **p < 0.01 in a One-way ANOVA followed by Dunnett’s test for multiple comparisons. ns, not significant.

**Figure 2:**
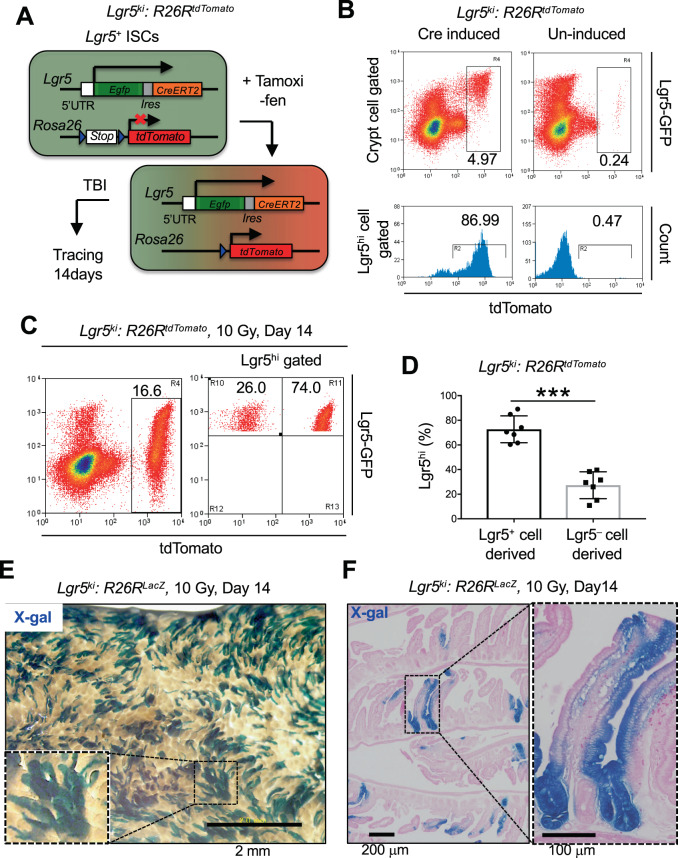
Lgr5-GFP^hi^ Cells Include the Cellular Source for Epithelial Regeneration. (**A**) Schematic of the lineage tracing of Lgr5^hi^ cells in *Lgr5*^*ki*^: *R26R*^*tdTomato*^ mice. (**B**) Representative FACS profiles of tdTomato expression in Lgr5^hi^ cells 24 h after tamoxifen injection (Cre induced, n = 4) or no injection (Un-induced, n = 2) in *Lgr5*^*ki*^: *R26R*^*tdTomato*^ mice. Plotted cells were gated on live EpCAM^hi^ cells (upper) or EpCAM^hi^ Lgr5^hi^ cells (lower). (**C**,**D**) Representative FACS profile of tdTomato expression in total crypt epithelial cells (**C**, left) and Lgr5^hi^ ISCs (**C**, right) 14 days after 10 Gy irradiation in *Lgr5*^*ki*^: *R26R*^*tdTomato*^ mice that had received a single injection of tamoxifen 24 h before irradiation. Plotted cells in **(C**) were gated on live EpCAM^hi^ cells. The average proportions with SD of tdTomato^+^ and tdTomato^–^ cells in the Lgr5^hi^ ISCs are shown in **D** (n = 7). ***p < 0.001 in a two-tailed Mann-Whitney test. (**E**,**F**) Representative macroscopic view and histologic analysis of X-gal-stained jejunum 14 days after 10 Gy irradiation in *Lgr5*^*ki*^: *R26R*^*LacZ*^ mice that had received a single injection of tamoxifen 24 h before irradiation (n = 3). Scale bars, 2 mm (**E**), 200 µm (**F**, left), 100 µm (F, right).

Within 48 h after exposure to 10 Gy TBI, the small intestinal crypts shrank, and the number of Ki67^+^ crypt epithelial cells was severely reduced as a result of transient mitotic arrest. The crypt shrinkage triggered the hyperproliferation of surviving radio-resistant cells, resulting in crypt enlargement at 1 week after TBI. By 2 weeks post-irradiation, the crypt architecture was recovered (Fig. 1A). Next, we examined the time-dependent changes in Lgr5^hi^ ISCs in the crypt after TBI using *Lgr5-eGFP-Ires-CreERT2* mice (hereafter *Lgr5*^*ki*^ mice). Most of the Lgr5^hi^ ISCs disappeared from the crypt within 48 h after irradiation, and then they gradually increased, and were completely restored by 2 weeks (Fig. 1A–C), implying that radio-resistant cells exist that have the potential to regenerate the Lgr5^hi^ ISC pool. To examine how much Lgr5^hi^ ISCs contribute to the recovery of the Lgr5^hi^ ISC pool, we crossed *Lgr5*^*ki*^ mice with a fluorescent reporter mouse line *Rosa26-lsl-tdTomato* (hereafter *Lgr5*^*ki*^: *R26R*^*tdTomato*^) and traced the fate of the Lgr5^hi^ ISCs after irradiation (Fig. 2A). In the *Lgr5*^*ki*^: *R26R*^*tdTomato*^ mice, the Lgr5^hi^ ISCs were exclusively labeled with tdTomato 24 h after a single injection of tamoxifen (Fig. 2B). Two weeks after irradiation, about 72.3 ± 10.6% of the recovered Lgr5^hi^ ISCs were positive for tdTomato, indicating that most of the regenerated Lgr5^hi^ ISCs originated from the previous Lgr5^hi^ ISCs (Fig. 2C,D). Consistent with this finding, another reporter line *Lgr5*^*ki*^: *Rosa26-lsl-LacZ* (hereafter *Lgr5*^*ki*^: *R26R*^*LacZ*^) mice, in which the Lgr5^hi^ cells express β-galactosidase after tamoxifen administration, showed that the Lgr5^hi^ ISCs had substantially supplied the villus epithelial cells observed 2 weeks after irradiation (Fig. 2E,F). Collectively, these results show that the Lgr5^hi^ ISCs include the cellular origin of the whole epithelium regeneration occurring upon TBI.

### Minor contribution of secretory progenitors to damage-induced epithelial regeneration

**Figure 3:**
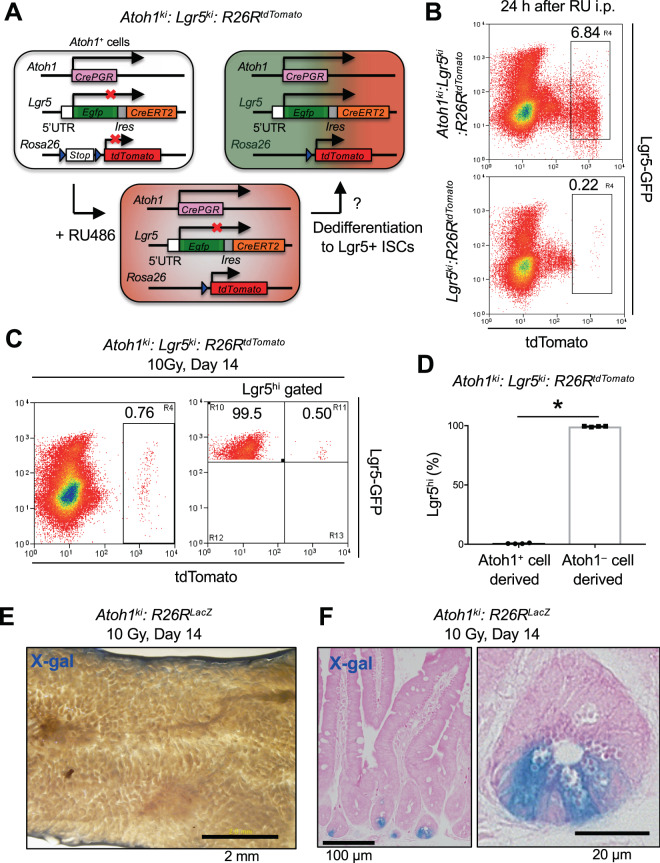
Contribution of Secretory Progenitors to Epithelial Regeneration after Irradiation Damage. (**A**) Schematic of the lineage tracing of Atoh1^+^ secretory cells in *Atoh1*^*ki*^: *Lgr5*^*ki*^: *R26R*^*tdTomato*^ mice. (**B**) Representative FACS profile of tdTomato expression in Atoh1^+^ cells 24 h after two injections of RU486 in either *Atoh1*^*ki*^: *Lgr5*^*ki*^: *R26R*^*tdTomato*^ mice (n = 4) or *Lgr5*^*ki*^: *R26R*^*tdTomato*^ mice (n = 2). Plotted cells were gated on live EpCAM^hi^ cells. (**C**,**D**) Representative FACS profile of tdTomato expression in total crypt epithelial cells (**C**, left) and in Lgr5^hi^ ISCs (**C** right) 14 days after 10 Gy irradiation in *Atoh1*^*ki*^: *Lgr5*^*ki*^: *R26R*^*tdTomato*^ mice that had received two injections of RU486 24 h and 16 h before irradiation. Plotted cells in C were gated on live EpCAM^hi^ cells. The average proportions with SD of tdTomato^+^ and tdTomato^–^ cells in Lgr5^hi^ ISCs are shown in (**D**) (n = 4). *p < 0.01 in a two-tailed Mann-Whitney test. (**E**,**F**) Representative macroscopic view and histologic analysis of X-gal-stained jejunum 14 days after 10 Gy irradiation in *Atoh1*^*ki*^: *R26R*^*LacZ*^ mice that had received two injections of RU486 24 h and 16 h before irradiation (n = 3). Note that Paneth cells were clearly identified by X-gal staining, providing evidence of the efficient induction of Cre–mediated recombination in secretory lineages in these mice. Scale bars, 2 mm (**E**), 100 µm (**F**, left), 20 µm (**F**, right).

Secretory progenitors can dedifferentiate into ISCs to contribute to the recovery of the ISC pool upon irradiation damage^7,11^. Thus, we next examined how much secretory progenitors contribute to the regeneration of the Lgr5^hi^ ISC pool and epithelial cells using the same intestinal injury model. The transcription factor Atoh1 specifically drives secretory lineage cell differentiation^18^. Therefore, to trace the fate of secretory progenitors after intestinal injury, we crossed *Atoh1-CrePGR* (*Atoh1*^*ki*^) mice with *Lgr5*^*ki*^*: R26R*^*tdTomato*^ mice (hereafter *Atoh1*^*ki*^*: Lgr5*^*ki*^*: R26R*^*tdTomato*^) (Fig. 3A). In naive *Atoh1*^*ki*^*: Lgr5*^*ki*^*: R26R*^*tdTomato*^ mice, two dose injection of RU486 successfully labeled the crypt Atoh1^+^ cells with tdTomato within 24 h (Fig. 3B). Compared with Lgr5^hi^ ISCs, the Atoh1^+^ cells exclusively expressed secretory cell-related genes, such as *Atoh1*, *Spdef*, *Lyz1*, *Defa6*, and *Muc2*, but not ISC marker genes, such as *Olfm4*, *Lgr5* and *Fstl1* (Fig. S1). The tdTomato labeled Atoh1-expressing cells included CD24^hi^ Side scatter^hi^ (SSC^hi^) Paneth cells (labeled cell frequency; 1.77 ± 0.63% in crypt epithelial cells, n = 5) and CD24^int^ SSC^lo^ secretory progenitors^7,19^ (labeled cell frequency; 1.59 ± 0.56% in crypt epithelial cells, n = 5) at a comparable frequency (Fig. S2A,B). As expected, CD24^int^ secretory progenitors prominently expressed *Mki67*, a proliferation marker gene, compared with non-proliferating CD24^hi^ Paneth cells (Fig. S2C). We then traced the fate of Atoh1^+^ cells after irradiation injury using *Atoh1*^*ki*^*: Lgr5*^*ki*^*: R26R*^*tdTomato*^ or *Atoh1*^*ki*^*:R26R*^*LacZ*^ mice, and found that these cells only minimally contributed to the Lgr5^hi^ ISCs and total epithelium observed 2 weeks after irradiation (Fig. 3C–E). Supporting the selective induction of Cre–mediated recombination in secretory lineages, Paneth cells were detected by X-gal staining at this time (Fig. 3F). Similar results were obtained even after injections of RU486 for five consecutive days, which label Atoh1^+^ crypt epithelial cells more efficiently than two times injections in these reporter mice^19^ (Fig. S3). Thus, the contribution of Atoh1^+^ secretory lineage cells to the recovery of ISCs and epithelial cells is much lower than that of the Lgr5^hi^ ISCs.

### Heterogeneity of surviving Lgr5-Derivatives after irradiation damage

**Figure 4:**
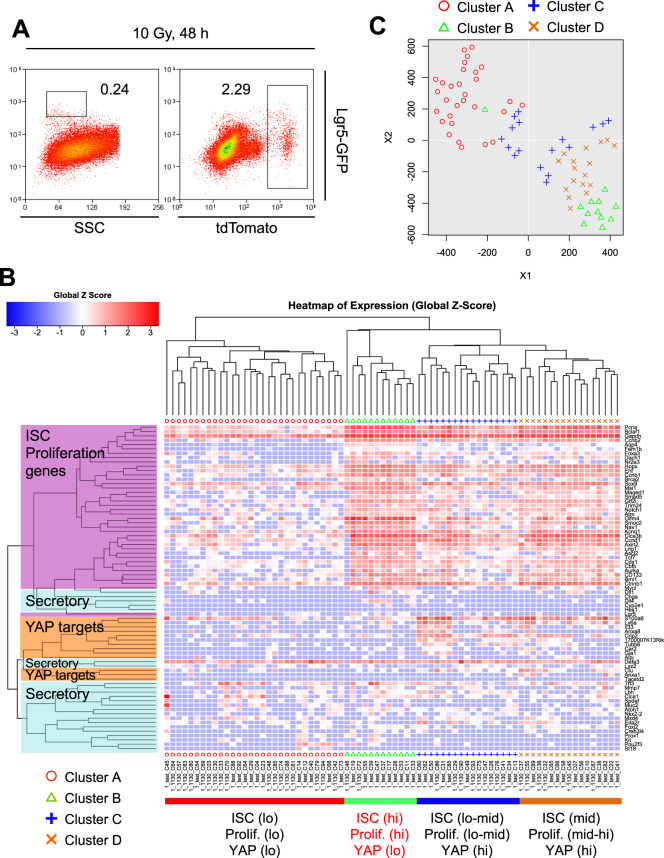
Surviving Lgr5-Derivatives after Irradiation Damage are Heterogeneous. *Lgr5*^*ki*^: *R26R*^*tdTomato*^ mice were exposed to 10 Gy irradiation 24 h after tamoxifen injection. Forty-eight h later, crypt epithelial cells were harvested, and scqRT-PCR analysis was performed. (**A**) Representative FACS profile of *Lgr5*^*ki*^: *R26R*^*tdTomato*^ mice 48 h after irradiation. Plotted cells were gated on live cells. (**B**) Heat map showing the hierarchical clustering of 76 single tdTomato^+^ cells 48 h after irradiation based on genes related to “ISC/Proliferation marker genes,” “YAP target genes,” and “Secretory cell related genes.” (**C**) tSNE plot of all samples.

To narrow down the cellular origin of epithelial regeneration, we focused on tdTomato^+^ cells in the damaged intestinal crypt of *Lgr5*^*ki*^: *R26R*^*tdTomato*^ mice at 48 h after irradiation (Lgr5-derivatives hereafter), the time of peak tissue damage and mitotic arrest. At this time, “phenotypic” Lgr5^hi^ ISCs had disappeared, while the Lgr5-derivatives (tdTomato^+^ cells) were still detected (Fig. 4A), suggesting that the Lgr5 expression had become silent. We then examined the gene expression profile of these surviving Lgr5-derivatives at the single-cell level using single-cell qRT-PCR (scqRT-PCR). We primarily focused on the expression of “ISC/proliferation marker” genes, because the cells poised to start regeneration will up-regulate or continue expressing these genes even in the damaged intestine. We also measured the expression of YAP target genes and some secretory lineage-associated genes, because intestinal damage activates YAP signaling to protect Lgr5^+^ ISCs from cell death^14^ and initiates dedifferentiation of secretory progenitors into ISCs^7^. Hierarchical clustering and tSNE analysis identified four clusters within the Lgr5-derivatives (Fig. 4B,C). The expression of all of these gene categories was lower in the Cluster A cells (Fig. S4). In contrast, the cells in Clusters B and D exhibited the highest expression of representative ISC marker genes such as *Aqp4, Smoc2*, and *Olfm4* and proliferation marker genes such as *Pcna, Ccnd1*, and *Ccnb1* (Fig. S4A,B). Based on these results, we concluded that Clusters B and D are most likely to contain the cell of origin for crypt epithelial regeneration. A fraction in Cluster B expressed some secretory cell marker genes such as *Dll4* or *Dll1* (Fig. S4C). On the other hand, the cells in Clusters C and D prominently expressed YAP target genes such as *Ly6a, S100a6, Tubb6, Anxa8*, *Crip2*, and *Clu* (Fig. S4D). YAP signal activation represses Wnt targets and ISC signatures in the intestinal epithelial cells^14^. In line with this, the expression levels of YAP target genes were inversely correlated well with those of Wnt target genes and ISC marker genes in each cluster (Fig. S4A,D). Therefore, diversity in the activation balance of YAP and Wnt signaling occurs among each Lgr5-derivatives after radiation injury.

### High epithelial regeneration potential in Sca1^–^Lgr5-Derivatives

**Figure 5:**
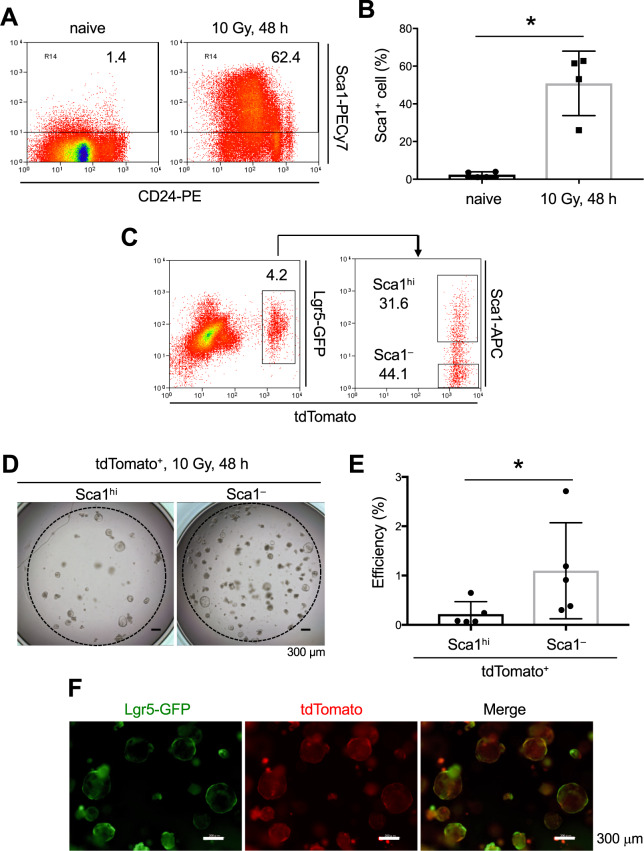
Sca1 Expression is Inversely Correlated with the Potential for Epithelial Regeneration in Lgr5-Derivatives. (**A**,**B**) Representative FACS profiles of the Sca1 and CD24 expressions of crypt epithelial cells (**A**) and the percentage of total crypt epithelial cells that were Sca1^+^ cells (**B**) (n = 4) before and 48 h after 10 Gy irradiation in WT mice. Plotted cells in A were gated on live EpCAM^hi^ cells. Data are means with SD of 4 mice. *p < 0.05 in a two-tailed Mann-Whitney test. (**C**) Representative FACS profiles of Sca1 expression of tdTomato^+^ Lgr5-derivatives in *Lgr5*^*ki*^: *R26R*^*tdTomato*^ mice 48 h after irradiation. Plotted cells were gated on live EpCAM^hi^ cells. (**D–F**) Representative images (**D**) and the efficiency (**E**) (n = 5) of organoids generated from Sca1^−^ or Sca1^hi^ Lgr5-derivatives. Lgr5^hi^ ISCs were regenerated from Sca1^–^ Lgr5-derivatives (**F**). Data are means with SD of 5 mice. *p < 0.05 in a two-tailed Mann-Whitney test. Scale bars, 300 µm.

*Ly6a*, which encodes Sca1, is a YAP target gene^14^ and cell surface expression of Sca1 is induced after epithelial damage in the small intestine^15,16^ and colon^17^. Among the Lgr5-derivatives, *Ly6a* was predominantly expressed in Clusters C and D but not in Clusters A and B (Figs. 4B and S4D). In this context, the Sca1 expression was negligible in the crypt epithelial cells in the steady state, whereas it was greatly up-regulated 48 h after irradiation (Fig. 5A,B), the time of peak intestinal damage. Since Sca1 is a cell-surface molecule, we separately isolated the Sca1^hi^ and Sca1^–^ Lgr5-derivatives at this time point and evaluated their ISC potential using organoid culture (Fig. 5C–F). Immediately after irradiation exposure, the production of EGF family proteins is markedly upregulated in the intestinal epithelial cells and surrounding stromal cells to promote epithelial regeneration^14^. To mimic the *in vivo* situation, we added epiregulin to the organoid culture of irradiated crypt epithelial cells. Notably, the organoid-formation efficiency of the Sca1^–^ fraction was substantially higher than that of the Sca1^hi^ population at 48 h after irradiation (Fig. 5D,E) and it became more remarkable at 3 days after irradiation (Fig. S5) In this context, Lgr5^hi^ ISCs were newly produced from the Sca1^–^Lgr5-derivatives in the organoids (Fig. 5F). These results indicated that the Sca1^–^Lgr5-derivatives containing Cluster B in the damaged intestine were enriched in the “cell-of-origin” for epithelial regeneration and that the contribution of Sca1-expressing Lgr5-derivatives in Cluster D to regeneration is low.

### Sca1^–^Lgr5-Derivatives are divided into two subpopulations

**Figure 6:**
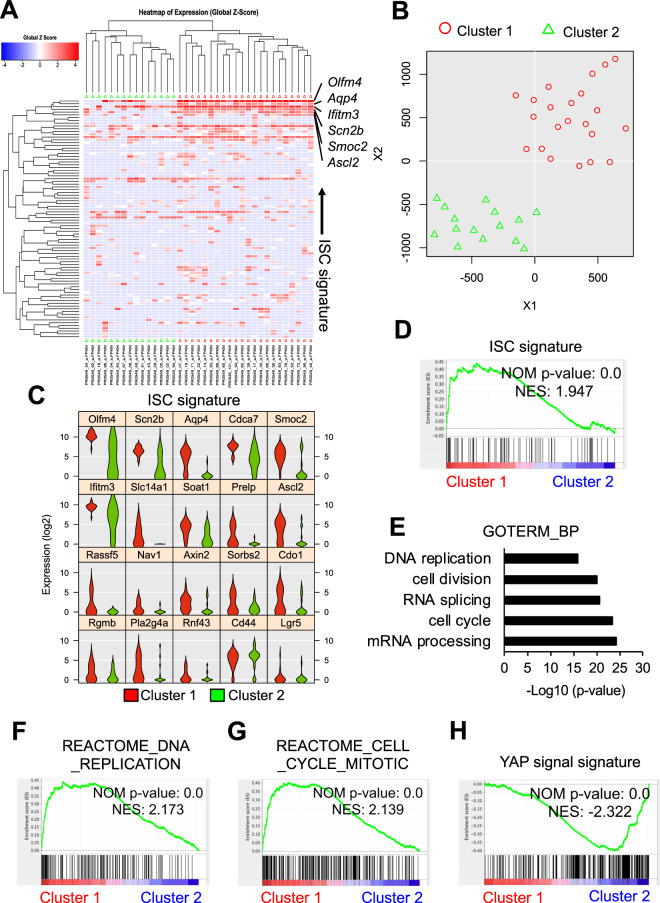
Single-Cell Gene Expression Analysis of Radioresistant Sca1^–^Lgr5-Derivatives. *Lgr5*^*ki*^: *R26R*^*tdTomato*^ mice were irradiated 24 h after tamoxifen injection. Sca1^–^ Lgr5-derivaetives (tdTomato^+^) were isolated 48 h after irradiation, and then single-cell RNA-sequencing was performed. (**A–C**) Hierarchical clustering (**A**), tSNE plot (**B**), and Violin plots for representative ISC signature genes (**C**) between Clusters 1 and 2 cells. (**D**) GSEA of the ISC signature (Supplementary Table S1) in Cluster 1 vs 2 cells. (**E**) GO biological processes enriched in the genes that were> 2-fold-upregulated in Cluster 1 versus Cluster 2 cells. (**F–H**) GSEA of the REACTOME DNA replication signature (**F**), REACTOME cell cycle and mitosis (**G**), and YAP signal signature (**H**, Supplementary Table S1) in Cluster 1 vs Cluster 2 cells.

Since the organoid-formation capacity was concentrated in the Sca1^–^ cell fraction within the Lgr5-derivatives in the damaged intestine, we further analyzed the heterogeneity of these cells by single-cell RNA sequencing. We applied Sca1^–^Lgr5-derivatives to the Fluidigm C1 system to generate cDNA at the single-cell level, and then obtained high-quality gene-expression profiles. Hierarchical clustering analysis based on the expression of ISC signature genes (Supplementary Table S1) revealed that these cells were grouped into two well-defined clusters, Cluster 1 and Cluster 2 (Fig. 6A,B). Although *Lgr5* expression was down-regulated in both Cluster 1 and Cluster 2 at this time point, other ISC-specific genes, such as *Olfm4, Scn2b, Aqp4, Cdca7, Smoc2, Ifitm3, Ascl2*, and *Axin2*, were predominantly expressed in Cluster 1 compared with Cluster 2 (Fig. 6C), indicating that the ISC potential was concentrated in Cluster 1 and further implying that Cluster 1 largely overlaps with Cluster B (Fig. 4). Consistent with these findings, Gene set enrichment analysis (GSEA)^20^ demonstrated that the ISC gene signature was highly represented in Cluster 1 compared with Cluster 2 (Fig. 6D).

### Characterization of Sca1^–^Lgr5-Derivatives expressing the ISC/proliferation signature

Cells poised to start regeneration up-regulate proliferation-related genes even in the damaged intestine. To further characterize the Cluster 1 cells, we performed Gene Ontology (GO) analysis using the transcriptional profiles that were more highly represented in Cluster 1 compared with Cluster 2. Notably, all of the top-ranked biological processes by this criterion were related to cell proliferation, including “mRNA processing,” “cell cycle,” and “DNA replication,” indicating that Cluster 1 cells are ready to divide (Fig. 6E). In line with these findings, GSEA revealed that gene signatures of “DNA replication,” “cell cycle mitotic,” and “MYC active pathway” were significantly enriched in Cluster 1 (Figs. 6F,G and S6A). Based on these results, we predicted that Cluster 1 enriches “cell-of-origin” for the regeneration of ISC pools and epithelial cells after irradiation injury. In this context, the Mex3a-expressing cells contain radioresistant quiescent ISCs^12^. However, the expression of *Mex3a* mRNA was not detected in the Sca1^–^Lgr5-derivatives (Fig. S7) suggesting that Cluster 1 and the Mex3a-expressing fraction are different populations. Furthermore, YAP-target genes (YAP signature) were underrepresented in Cluster 1 compared with Cluster 2 in scRNA-seq data (Figs. 6H and S8). In this context, *Clu*-expressing revival stem cells (revSCs) transiently expand in a YAP dependent manner after radiation damage and regenerate Lgr5^+^ ISCs and a functional epithelium^15^. However, both Cluster 1 and 2 cells expressed low Yap target genes including *Clu* (Fig. S8) and high stem cell/proliferation marker genes, suggesting that they are different from revSCs.

To evaluate the proliferation status of Cluster 1, we generated a list of cell cycle-promoting genes in the steady-state Lgr5^hi^ ISCs by identifying genes that were expressed more than 2-fold higher in Lgr5^hi^ ISCs than in Paneth cells, a type of non-proliferating crypt epithelial cells, from two different data sources (GSE25109, GSE39915). By comparing these cell cycle-promoting genes with the genes prominently expressed in Cluster 1 versus 2, we found 986 genes that were uniquely expressed in Cluster 1 (Fig. S6B). Notably, GO analysis of these unique genes revealed that biological processes related to cell proliferation were ranked in the top 5 (Fig. S6C). These results indicated that, upon tissue injury, the expression profile of cell cycle-related genes in surviving Lgr5^+^ ISCs dynamically switched to regeneration mode that rapidly replenish the lost ISC pool after irradiation injury, while it is not mediated by YAP signal activation.

### Preservation of the ISC potential in CD81^hi^ Sca1^–^ cells of the damaged intestine in WT mice

**Figure 7:**
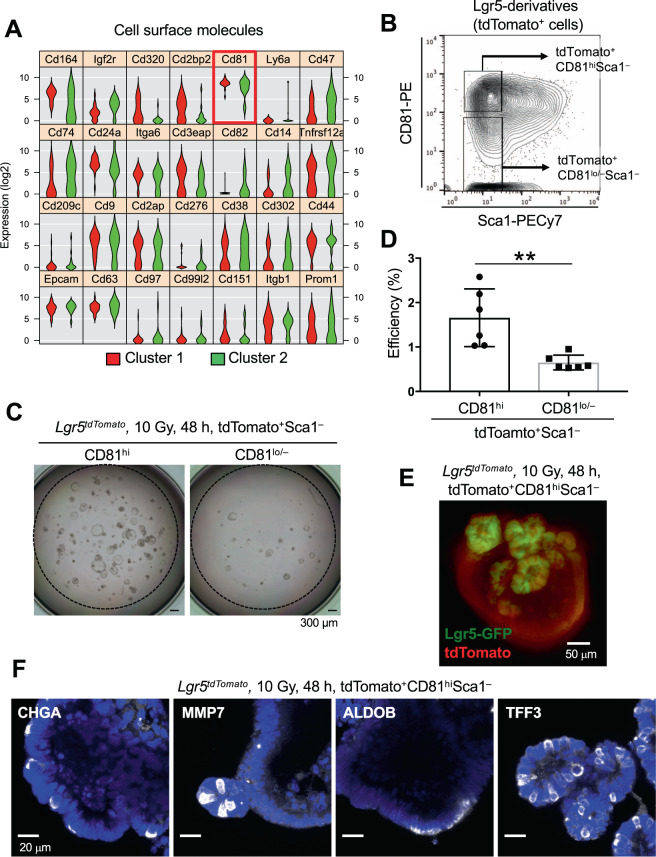
High CD81 Expression Distinguishes Cluster 1 in Radioresistant Lgr5-derivatives. (**A**) Violin plots for representative cell-surface molecules that were differentially expressed (p < 0.05) in Clusters 1 and 2 of Sca1^–^Lgr5-derivatives 48 h after 10 Gy irradiation. (**B**) Representative FCM profile of the Sca1 and CD81 expression of Sca1^–^Lgr5-derivatives. Plotted cells were gated on live tdTomato^+^Lgr5-derivatives. (**C**,**D**) Representative images (**C**) and the efficiency (**D**) (n = 6) of organoids generated from CD81^hi^ or CD81^lo/−^ Sca1^–^Lgr5-derivatives, 48 h after 10 Gy irradiation. **, *p* < 0.01 in a two-tailed Mann-Whitney test. Scale bars, 300 µm. (**E**) ISC regeneration from CD81^hi^Sca1^–^Lgr5-derivatives in organoid culture. CD81^hi^Sca1^–^Lgr5-derivatives were isolated from *Lgr5*^*tdTomato*^ mice 48 h after 10 Gy irradiation. Representative organoid image of Lgr5-GFP and tdTomato expression after 9 days of culture. Scale bar, 50 µm. (**F**) Multipotency of CD81^hi^Sca1^–^Lgr5-derivatives. Representative images of organoids derived from CD81^hi^Sca1^–^Lgr5-derivatives stained for enteroendocrine cells (CHGA), Paneth cells (MMP7), enterocytes (ALDOB) and goblet cells (TFF3). Nuclear staining was performed with DAPI (Blue). Scale bars, 20 µm.

**Figure 8:**
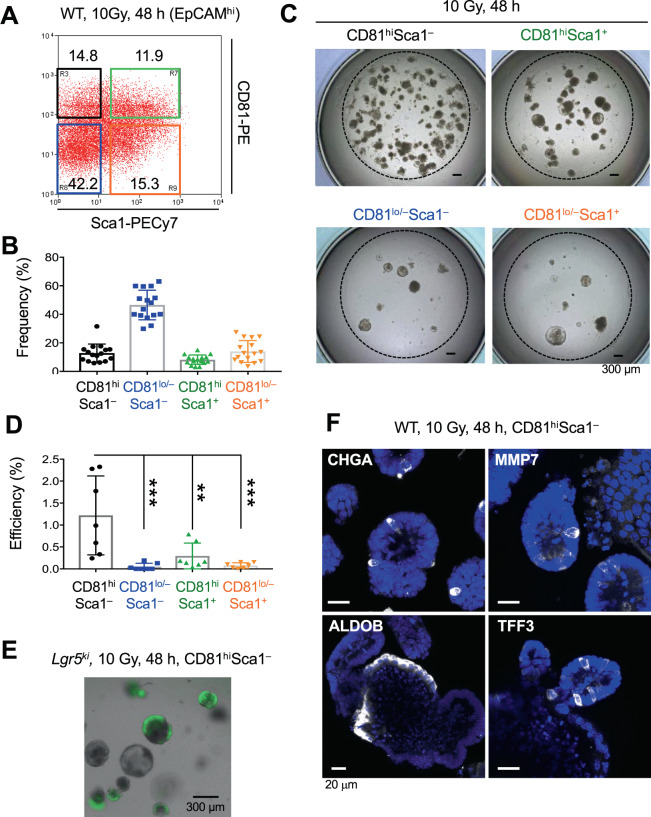
CD81 Is a Robust Cell Marker for Identifying Radioresistant ISCs in Damaged Epithelium. (**A**) Representative FCM profile of the Sca1 and CD81 expression of crypt epithelial cells of WT mice 48 h after 10 Gy irradiation. Plotted cells were gated on live EpCAM^hi^ cells. (**B**) Frequency of the subpopulations of crypt epithelial cells in (**A**) (n = 16). (**C**,**D**) Representative images (**C**) and the efficiency (**D**) (n = 7) of organoids generated from CD81^hi^ Sca1^−^, CD81^hi^ Sca1^+^, CD81^lo/-^ Sca1^-^, or CD81^lo/-^ Sca1^+^ cells, 48 h after 10 Gy irradiation in WT mice. **p < 0.01, ***p < 0.001, in a One-way ANOVA followed by Dunnett’s test for multiple comparisons. Scale bars, 300 µm. (**E**) Lgr5^hi^ ISCs were regenerated from CD81^hi^ Sca1^−^ cells isolated from *Lgr5*^*ki*^ mice 48 h after 10 Gy irradiation. Scale bar, 300 µm. (**F**) Multipotency of CD81^hi^ Sca1^-^ crypt epithelial cells in irradiation-damaged WT mice. Representative images of organoids derived from CD81^hi^Sca1^−^ cells stained for enteroendocrine cells (CHGA), Paneth cells (MMP7), enterocytes (ALDOB) and goblet cells (TFF3). Nuclear staining was performed with DAPI (Blue). Scale bars, 20 µm.

To validate the stem cell potential of Cluster 1 by organoid formation assay, we first searched for cell-surface molecules to isolate the fraction containing Cluster 1 from the damaged intestine of genetically unmodified WT mice. We found that the expression of some surface molecules, such as *Cd164*, *Cd320*, and *Cd81* was significantly higher in Cluster 1 than Cluster 2 (*Cd164: p* = *0.0029*, *Cd320: p* = *0.0068*, *Cd81: p* = *0.0358*, One-way ANOVA*)*. In contrast, the expression level of *Cd44* and *Prom1* (also known as *Cd133*), both of which are well known ISC markers, could not distinguish between Cluster 1 and Cluster 2 (*Cd44: p* = *0.5563*, *Prom1: p* = *0.9832*, One-way ANOVA) (Fig. 7A). Among these molecules, we selected *Cd81*, which encodes CD81, a member of the tetraspanin family^21^, as a primary candidate because it had the highest expression level, a requirement for isolation by cell-sorting (Fig. 7A). Although the expression of tetraspanin family molecules on ISCs has not been reported, they are uniquely expressed on other tissue stem cells both in mice and in humans^22–24^. In this context, we found that all Lgr5^hi^ ISCs highly expressed CD81, while its expression was significantly lower in most Lgr5^lo^ progenitors in the steady state (Fig. S9A,B). Consistent with these observations, immunohistochemical analysis showed that CD81 was predominantly detected in the crypt bottom epithelial cells including Lgr5^+^ ISCs, but not in villi (Fig. S9C). Some immune cells in the lamina propria of the villi also expressed CD81 (Fig. S9C), as previously reported^25–28^. Thus, we next performed co-immunostaining of CD81 and Sca1 in intestinal tissue sections of 10 Gy irradiated mice in addition to naïve mice. Similar to FCM analysis (Figs. 8A,B and S9A,B), CD81 was distinctly expressed on the crypt epithelial cells both in naïve and in irradiation exposed WT mice, whereas Sca1 expression was detected only after irradiation exposure. Of note, CD81^hi^Sca1^–^ cells are preferentially localized at the crypt bottom of the damaged intestine (Fig. S10). To be precise based on the violin plot of CD81 in Fig. 7A, CD81^hi^ cells likely contain most Cluster 1 cells and a portion of Cluster 2 cells expressing CD81 at highly levels. On the other hand, most of the CD81^lo^Sca1^–^Lgr5-derivatives are Cluster 2. Thus, CD81 is a useful marker to enrich Cluster 1 cells. To directly demonstrate this, we sorted CD81^hi^ or CD81^lo/–^ cells within Sca1^–^Lgr5-derivatives at 48 h after irradiation (Fig. 7B) and evaluated their organoid formation efficiency. Notably, CD81^hi^Sca1^lo/–^ Lgr5-derivatives revealed a significantly higher organoid formation efficiency than CD81^lo/–^Sca1^–^ Lgr5-derivatives, indicating that CD81 is indeed a useful molecular marker to enrich and evaluate Cluster 1 cells (Fig. 7C,D). Notably, these organoids contained all intestinal lineages, including Paneth cells, Goblet cells, Endocrine cells, enterocytes, as well as Lgr5^+^ ISCs, indicating that CD81^hi^Sca1^–^ Lgr5-derivatives have multilineage differentiation potential and no lineage differentiation bias (Fig. 7E,F). Next, based on the expression levels of CD81 and Sca1, we separately isolated four different fractions —CD81^hi^ Sca1^−^ (Cluster 1 and part of Cluster 2), CD81^lo/−^ Sca1^−^ (remaining part of Cluster 2), CD81^hi^ Sca1^+^, and CD81^lo/–^ Sca1^+^ from WT mice 48 h after irradiation (Fig. 8A,B) and examined their stem-cell potential by organoid-formation assay. As expected, a prominent organoid-formation capacity was detected in the CD81^hi^ Sca1^–^ cell fraction containing Cluster 1 (Fig. 8C,D), suggesting that Cluster 1 enriches the source for regeneration in the damaged intestine. Again, Lgr5-expressing ISCs and multilineage cells were visually reconstituted in organoids from CD81^hi^Sca1^-^ cells prepared from *Lgr5*^*ki*^ mice and WT mice after irradiation (Fig. 8E,F).

## Discussion

Although several groups have tried to identify the cellular source of intestinal epithelial regeneration after damage using genetic labeling and the tracing of distinct cell types, such approaches cannot assess how much each population contributes to overall epithelial regeneration or exclude the possibility that other cellular sources exist. To clarify the entire scheme of damage-induced intestinal epithelial regeneration, including the cells of origin, it is necessary to investigate surviving crypt epithelial cells for their functional stem cell potential along with comprehensive gene expression profiling.

In this study, we characterized the heterogeneity of epithelial stem cells in damaged intestinal crypts using a combination of lineage tracing, single-cell gene expression analysis, and organoid-formation assays. Most of the “cells-of-origin” were included in the Lgr5-derivatives, consistent with a previous report showing that Lgr5^+^ ISCs are essential for intestinal epithelial regeneration after irradiation injury^13^. However, the Lgr5^+^ cells have substantial heterogeneity and include slowly dividing populations, such as Mex3a^+^ cells^12^ and label retaining cells (LRCs)^11^. In addition, Dll1^+^ secretory precursor cells are the immediate progeny of Lgr5^+^ ISCs and are slowly cycling cells within the crypt epithelium^7^. Because all of these cells are relatively resistant to DNA damage, they have been considered as possible “cells-of-origin” for the epithelial regeneration after genotoxic injury. By genetic lineage tracing, Dll1^+^ epithelial cells and LRCs were previously shown to be precursors committed to secretory epithelial cells in the steady state, and to dedifferentiate into ISCs and participate in epithelial regeneration upon intestinal damage^7,11^. Furthermore, differentiated secretory epithelial cells, including enteroendocrine cells^8^ and Paneth cells^9,10^, also regain stem cell properties during intestinal damage. Consistent with these findings, a portion of colonic crypt epithelial cells expressing *Atoh1*, a transcription factor essential for secretory lineage differentiation, contributed to epithelial regeneration in a dextran sodium sulfate (DSS) colitis model^19^. However, our lineage-tracing data suggested that Atoh1^+^ undifferentiated secretory lineage cells are at least not substantially involved in the epithelial regeneration after irradiation injury in the small intestine. Supporting this conclusion, the intestinal epithelium is fully reconstituted after irradiation injury in *Atoh1-*deficient mice^29^. Our results also showed that a fraction of Cluster B expresses *Dll4* or *Dll1* (Fig. S4C), implying the involvement of this fraction as secretory precursors in ISC recovery and epithelial regeneration. In this respect, we emphasize that our results do not contradict the presence of intestinal epithelial cell plasticity, i.e., Dll1^+^ secretory precursors dedifferentiate into ISCs after irradiation^7^.

Other cell types also contribute to the epithelial regeneration after irradiation- and chemotherapy-induced injury^12,15^. Mex3a^+^ cells do not show any biased commitment to secretory cell lineages in the steady state^12^. Because of their slow-proliferation status, Mex3a-expressing ISCs survive 2 days after irradiation exposure^12^. However, our single-cell RNA-sequencing data showed that surviving Cluster 1 cells did not express *Mex3a* mRNA (Fig. S7), suggesting that Cluster 1 is a distinct population from Mex3a^+^ cells. After intestinal damage by irradiation, YAP signal dependent revSCs transiently expand and regenerate a functional epithelium^15^. According to the gene expression profiles, *Clu*-expressing revSCs are distinct from our Cluster 1 and 2, which have high expression of cell cycle related genes and ISC marker genes, and low expression of YAP target genes. In the scRNA-seq data using crypt epithelial cells after irradiation, the revSCs and our Cluster 1 and 2 appear to be classified as different populations^15^. Thus, our work and the work of Ayyaz *et al*. are mutually complementary to explain complete picture of radioresistant ISCs. Depending on the type and degree of intestinal damage, a variety of cell types could be synchronistically involved in the epithelial regeneration.

In this study, we newly discovered that CD81, a tetraspanin family protein, is prominently expressed on mouse ISCs in both the steady-state and damaged intestine. CD81 is a useful indicator for radioresistant ISCs, particularly in the damaged intestine where the Lgr5 expression became silent. Although this is the first report of CD81 expression on ISCs, other tetraspanin family molecules are preferentially and uniquely expressed on other tissue stem cells in mice and humans, including CD9 on mouse hematopoietic stem cells^22^, tetraspanin KAI/CD82 on stem cells of human fetal and adult skeletal muscle^24^, and tetraspanin 8 on mouse mammary stem cells^23^. It will be interesting to examine whether the surface expression of tetraspanin family molecules is useful for identifying stem cells in various steady-state and damaged tissues and to further elucidate the biological function of tetraspanin family members in the maintenance, survival, and activation of tissue stem cells. In addition, a risk for colorectal cancer (CRC) recurrence is associated with the expression of ISC-specific genes, including *Lgr5*, *Ascl2*, and *Ephb2*, in the human primary tumors^30^. In this context, tetraspanin proteins are known to have supportive roles in human cancer, for example, in tumor growth, morphology, invasion, and metastasis^31^. Future investigations will uncover a panel of available tetraspanins, including CD81, that can serve as markers for cancer stem cells in diverse epithelial tissues.

Tissue regeneration is a complex process involving the coordination of diverse cell types and molecules. Thus, it is impossible to obtain the entire scheme of tissue regeneration, including the cellular origin, by classic genetic labeling and lineage tracing alone. Our study integrating single-cell transcriptome analysis and organoid formation assays was effective for uncovering the heterogeneity of surviving epithelial cells and identifying the “real” cellular source of epithelium regeneration after tissue injury. Using this approach, we identified the crucial cellular source for tissue regeneration in the damaged intestine, and demonstrated that the contribution of epithelial cell plasticity is relatively minor. Given that organoid culture is now available for diverse tissues^32^, our findings and approach might be applied to identify the cellular origin for a variety of tissue-regeneration systems in injured and diseased conditions.

## Methods

### Mice and ethics statement

*Lgr5-EGFP-Ires-CreERT2* (*Lgr5*^*ki*^, B6.129P2- *Lgr5* ^*tm1(cre/ERT2)Cle*^ /J; JAX mice #008875), *Rosa-lsl-tdTomato* (*R26R*^*tdTomato*^, B6.Cg-Gt(ROSA)26Sortm9/(CAG-tdTomato)Hze/J; JAX#007909), and *Rosa-lsl-LacZ* (*R26R*^*Lacz*^, B6.129S4-Gt(ROSA)26Sor^tm1Sor^/J; Jax #003474) mice were obtained from Jackson Laboratory. For the irradiation injury of intestinal epithelium, mice were exposed to 10 Gy TBI (Acrobio, RX-650). For lineage-tracing experiments using *Lgr5*^*ki*^: *R26R*^*tdTomato*^ or *Lgr5*^*ki*^: *R26R*^*LacZ*^ mice, 100 mg/kg body weight (BW) of tamoxifen (Sigma) was injected intraperitoneally 24 h before irradiation.

For lineage-tracing experiments using *Atoh1*^*ki*^: *Lgr5*^*ki*^: *R26R*^*tdTomato*^ or *Atoh1*^*ki*^: *R26R*^*LacZ*^ mice, 2 doses of 100 mg/kg BW of RU486 (Sigma) was injected intraperitoneally at 24 h and 16 h before irradiation. In another experimental setting (Fig. S4), *Atoh1*^*ki*^: *R26R*^*LacZ*^ mice were injected RU486 intraperitoneally for 5 consecutive days before irradiation. All experiments with mice were approved by the Institutional Animal Care Committee of Tokyo Medical and Dental University and were performed in accordance with Tokyo Medical and Dental University guidelines.

### Crypt isolation and sorting

Crypts were isolated from the small intestine as described previously^33^ with some modifications. To prepare single cells, isolated crypts were incubated with TrypLE Express (Invitrogen) at 37 °C for 25 min with gentle pipetting, and then washed with PBS(−) containing 10% FCS and filtered through 70-µm mesh to collect the cells. Dissociated crypt epithelial cells were stained with antibodies. Epithelial-cell subpopulations were sorted using a MoFlo cell sorter (Beckman Coulter) or a BD FACS Aria III (BD Bioscience).

### X-gal staining

For X-gal staining, intestines were incubated with a fixation solution [2% PFA, 0.2% glutaraldehyde, and 0.02% NP40 in PBS(−)] on ice for 1 h. The fixation solution was removed, and the intestines were washed twice in PBS(−). Next, the tissues were soaked in X-gal substrate solution [5 mM K_3_Fe(CN)_6_, 5 mM K_4_Fe(CN)_6_, 2 mM MgCl_2_, 0.02% NP40, and 1 mg/ml X-gal in PBS(−)] and incubated in the dark at room temperature overnight. The substrate was removed, and the tissues were washed twice in PBS(−) for 20 min at room temperature. The tissues were fixed overnight in 10% formalin at 4 °C, embedded in paraffin, and sectioned.

### Immunohistochemistry

The proximal jejunal tissue was fixed in 4% PFA for 1 h at 4 °C, then the tissue was cryo-protected in 15% sucrose for 4 h followed by 30% sucrose overnight at 4 °C. For the immunostaining, frozen sections of the small intestine were blocked with 10% BSA/PBS, then with an Avidin/Biotin-blocking kit (Vector Labs) if required. The sections were stained with primary antibodies for 1 h at 37 °C or overnight at 4 °C. After washing with 0.05% Triton X-100 PBS(−), the sections were incubated with secondary antibodies or fluorescence-conjugated streptavidin for 1 h at 37 °C. Nuclear staining was performed using DAPI and sections were mounted with Fluoromount-G (Southern Biotech). Microscopic images were obtained using Leica SP8 confocal microscope.

### qRT-PCR

Total RNA was extracted using an RNeasy Mini Kit (Qiagen). First-strand cDNA was synthesized from the total RNA using SuperScript III (Life Technologies). Real-time PCR was performed using SYBR green (Roche) and a LightCycler 480 instrument, and RNA levels were calculated using the ∆CT method with normalization to *Hprt* expression. The primers used for qRT-PCR are listed in Supplementary Table S2.

### Single-cell qRT-PCR

FCM-sorted single crypt epithelial cells were introduced into the cell input well of the C1 Array Integrated Fluidic Circuit (IFC) (5–10 µm). Single cells captured on the IFC were microscopically inspected with a Keyence BZ-X700 to determine which C1 capture sites contained only a single cell. Reverse transcription and specific-target amplification were performed using reagents of the Single Cells-to-Ct kit (Life Technologies), C1 Single-Cell Auto Prep Modules Kit (Fluidigm), and pooled primers (Delta Gene, 500 nM). qPCR of these preamplified products was performed using 96.96 Dynamic Arrays on a BioMark HD System Fluidigm), according to the manufacturer’s instructions, and analyzed with the SINGuLAR Analysis Toolset (Fluidigm). All oligonucleotide sequences are shown in Supplementary Table S3.

### Organoid culture

Organoid-formation assays were performed as previously described^34^ with some modifications. Sort-purified crypt epithelial cells were mixed into Matrigel (3000–5000 cells/10 µl) containing 1 µM Jagged1 peptide, and then 10 µl of the sorted cell/Matrigel mixture was seeded into each well of a 96-well plate. The Matrigel was allowed to solidify for 15 min in a 37 °C incubator and then was overlaid with 100 µl culture medium containing advanced DMEM/F12 supplemented with penicillin/streptomycin, 10 mM HEPES, Glutamax, 1× N2, 1× B27 (all from Invitrogen), 1 µM N-acetylcysteine (Sigma), 50 ng/ml EGF, 100 ng/ml Noggin (Peprotech), 10% RspoI-conditioned medium (culture supernatant of the 293T-HA-RspoI-Fc cell line, provided by Calvin Kuo of Stanford University), 500 ng/ml Epiregulin, 10 µM Y-27632 (first 2 days, Nacalai Tesque), and 3 µM CHIR-99021 (Axon Medchem) (hereafter referred to as NER + CH + Ereg Medium), and the cells were cultured for 7 days. The medium was changed every other day. Microscopic images of the organoid cultures were obtained with a Keyence BZ-X700.

### Wholemount organoid staining

FCM sorted Sca1^–^CD81^hi^ cells from irradiated WT mice or Sca1^–^CD81^hi^ tdTomato^+^ cells from irradiated *Lgr5*^*ki*^: *R26R*^*tdTomato*^ mice were cultured for 6 days in NER + CH + Ereg Medium, after which the medium was changed to general NER medium and cultured for another 2 days. Organoids were released from Matrigel using Cell Recovery Solution (Corning) and were fixed in 4% paraformaldehyde for 60 min at room temperature. After washing, organoids were permeabilized with 0.2% Triton X-100 in PBS for 60 min at room temperature. The organoids were further blocked using 1% BSA/PBS for 60 min at room temperature, followed by primary-antibody reactions at 4 °C overnight. The organoids were then washed three times with 1% BSA/PBS and stained with secondary antibodies containing DAPI for 2 h at room temperature with protection from light. Stained organoids were suspended in Fluoromount-G (Southern Biotech) and mounted onto a 8 well glass bottom chamber. Organoid images were captured using Leica SP8 confocal microscope.

### Single-cell RNA sequencing and gene-expression quantification

FCM-sorted single crypt epithelial cells were introduced into the cell input well of the C1 Single-Cell Auto Prep System (5–10 µm). Single cells captured on the IFC were microscopically inspected with a Keyence BZ-X700 to determine which C1 capture sites contained only a single cell. Reverse transcription and cDNA preamplification were performed using a SMARTer Ultra Low RNA kit (Clontech). Sequencing libraries were prepared using the Nextera XT DNA Sample Preparation Kit and the Nextera Index Kit (Illumina), according to the manufacturer’s instructions. Libraries from 39 single cells were pooled and sequenced on Illumina HiSeq. 2500 using paired-end 100-base reads. The preparation and sequencing of cDNA libraries and the extraction of multiple expression data were performed by Takara Bio, Inc. Further data analysis was performed using the SINGuLAR software Toolset (Fluidigm).

### Antibodies

The following antibodies (BioLegend) were used for flow cytometry: Biotin-conjugated anti-mouse Sca1 (D7), BV421-labeled anti-mouse Sca1 (D7), APC-labeled anti-mouse EpCAM (G8.8), FITC-labeled anti-mouse EpCAM (G8.8), PE-labeled anti-mouse CD24 (M1/69), Biotin-conjugated anti-mouse CD24 (M1/69), Biotin-conjugated anti-mouse CD81 (Eat2), Streptavidin-APC, and Streptavidin-PECy7. For immunofluorescence staining of sections, the following antibodies were used: Biotin-conjugated anti-mouse CD81 (Eat2, BioLegend), PE-labeled anti-mouse CD81 (Eat2, BioLegend), Purified anti-mouse Sca1 (e13-161.7, BioLegend), Biotin-conjugated anti-mouse Sca1 (177228, R&D Systems), Streptavidin-Alexa647 (BioLegend), Alexa488-labeld anti-Rat IgG (Jackson). Dead cells were excluded by propidium iodide (Sigma) or Zombi NIR (BioLegend) staining.

### Bioinformatics

To analyze the GO term enrichment for biological process and cellular components in the selected genes, we used the DAVID Bioinformatics Resources 6.8 (http://david.abcc.ncifcrf.gov/ home.jsp)^35^. Gene-set enrichment analysis (GSEA)^20^ was performed using microarray data combined with the GSEA v2.0.13 software (Broad Institute, http://www.broadinstitute.org/gsea/msigdb/index.jsp). Gene sets were obtained from the Molecular Signatures Database (MSigDB) v4.0 available at the GSEA website or from previously reported microarray data (GSE33949 for “ISC signature,” GSE66567 for “YAP signal signature). The number of permutations was set to 1000. Gene sets with nominal *p* values <0.05 are considered statistically significant.

### Statistical analysis

Statistical analysis was performed using the two-tailed Mann-Whitney test or Welch’s t test for comparing 2 groups and One-way ANOVA followed by Dunnett’s test for comparing multiple groups. A *p* value <0.05 is considered statistically significant.

## Supplementary information

Supplementary Information.

## Data Availability

The scRNA-seq data have been deposited in the Gene Expression Omnibus with accession numbers GSE146783.
