# Supplementary material for: Characterization of radioresistant epithelial stem cell heterogeneity in the damaged mouse intestine
Source: Sci Rep. 2020 May 22;10:8308. doi: 10.1038/s41598-020-64987-1 (PMC7244543; doi:10.1038/s41598-020-64987-1)
Supplement: Supplementary file 1 — Supplementary Information. [file 41598_2020_64987_MOESM1_ESM.pdf]

Characterization of radioresistant epithelial stem cell heterogeneity in the damaged mouse intestine

Taku Sato[1,2,5], Miwako Sase[1,3,5], Shun Ishikawa[1], Mihoko Kajita[1], Jumpei Asano[1], Toshiro Sato[4], Yoshiyuki Mori[3], and Toshiaki Ohteki[1,6,*]

[1]Department of Biodefense Research, Medical Research Institute, Tokyo Medical and Dental University, Tokyo 113-8510, Japan.
[2]PRESTO, Japan Science and Technology Agency, Saitama 332-0012, Japan. [3]Department of Dentistry, Oral and Maxillofacial Surgery, Jichi Medical University, Tochigi 329-0498, Japan.
[4]Department of Gastroenterology, Keio University School of Medicine, Tokyo 160-8582, Japan.
[5]These authors contributed equally: Taku Sato, Miwako Sase
[6]Lead contact.
*Correspondence: ohteki.bre@mri.tmd.ac.jp

Supplementary Figure S1. Sato et al.

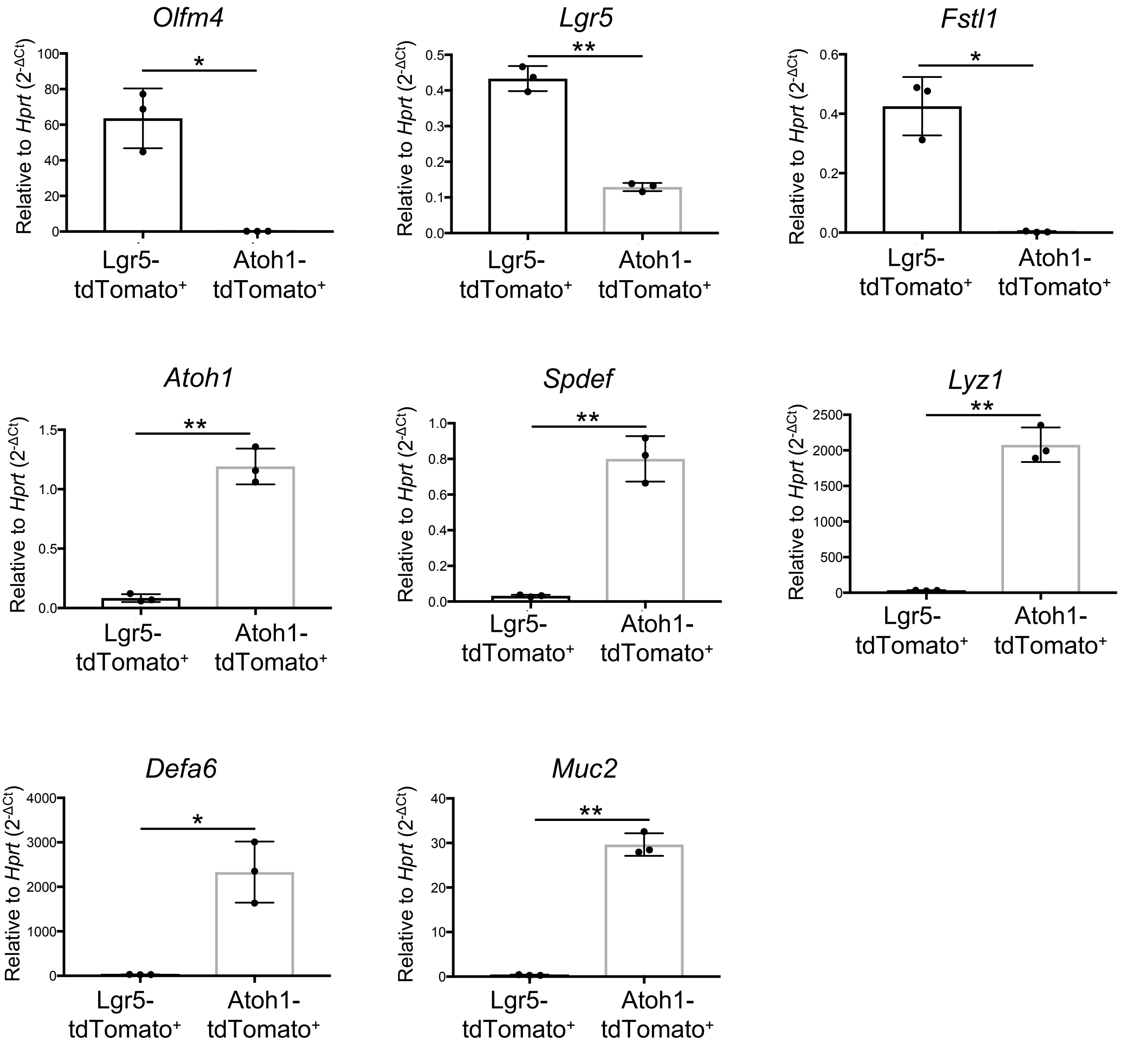

Figure S1. Gene expression profiles of Lgr5^{+}cells and Atoh1^{+} cells, Related to Figure 2. tdTomato^{+} crypt epithelial cells were isolated from either *Lgr5^{ki}*: *R26R^{tdTomato}* (Lgr5-tdTomato^{+}, n = 3) or *Atoh1^{ki}*: *Lgr5^{ki}*: *R26R^{tdTomato}* (Atoh1-tdTomato^{+}, n = 3) mice 24 h after tamoxifen (1 dose) or RU486 (2 dose) administration. The relative mRNA expression levels of ISC marker genes (*Olfm4*, *Lgr5*, *Fstl1*) and of secretory cell marker genes (*Atoh1, Spdef, Lyz1, Defa6, Muc2*) in tdTomato^{+} crypt epithelial cells from each mouse line were assessed by qRT-PCR. Data shows mean ± SD of three mice. Each dot represents the data from an individual mouse. *, $p < 0.05$, **, $p < 0.01$ in a Welch's t test.

Supplementary Figure S2. Sato et al.

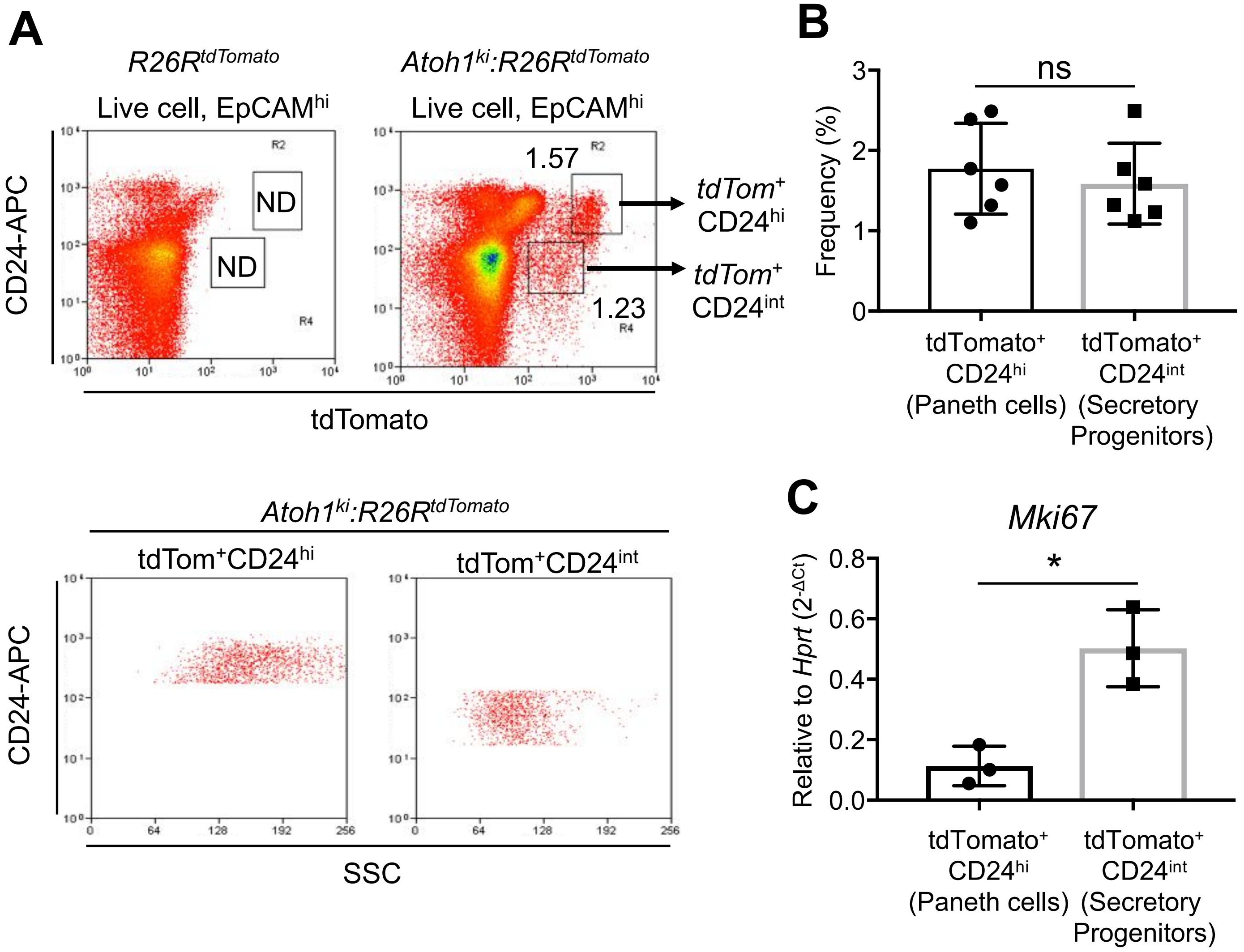

Figure S2. Atoh1^{+}CD24^{int} undifferentiated secretory cells were labeled by RU486 administration in *Atoh1-CrePGR:Rosa-lsl-tdTom* reporter mice, Related to Figure 3. *Atoh1*^{ki}: *R26R*^{tdTomato} or *R26R*^{tdTomato} mice were injected with 2 doses of RU486, and crypt epithelial cells were harvested 24 h later. (**A**) Representative FACS profiles of *R26R*^{tdTomato} mice (upper left, n = 2) or *Atoh1*^{ki}: *R26R*^{tdTomato} mice (upper right, n = 5). Plotted cells were gated on live crypt epithelial cells. Note that some CD24^{int} undifferentiated cells indeed expressed *Atoh1* and were detected as tdTomato^{+} cells (1.59 ± 0.56 %). The tdTomato^{+}CD24^{hi} cells are Paneth cells because of their highly SSC intensity cells (lower left) compared with undifferentiated Atoh1^{+}CD24^{int} secretory progenitors (lower right). ND: not detected. (**B**) The frequency of either tdTomato-labeled Paneth cells or tdTomato-labeled secretory progenitors. Data show means ± SD of six mice. Each dot represents the data from an individual mouse. ns: not significant in a two-tailed Mann-Whitney test. (**C**) The relative mRNA expression level of the proliferation marker *Mki67* was assessed by qRT-PCR in tdTomato^{+}CD24^{hi} cells (Paneth cells) or CD24^{int} cells (secretory progenitor cells). Data shows mean ± SD of three independent experiments. Each dot represents an individual experiment (pooled samples from 1-2 mice/experiment). *, p<0.05 in a Welch's t test.

Supplementary Figure S3. Sato et al.

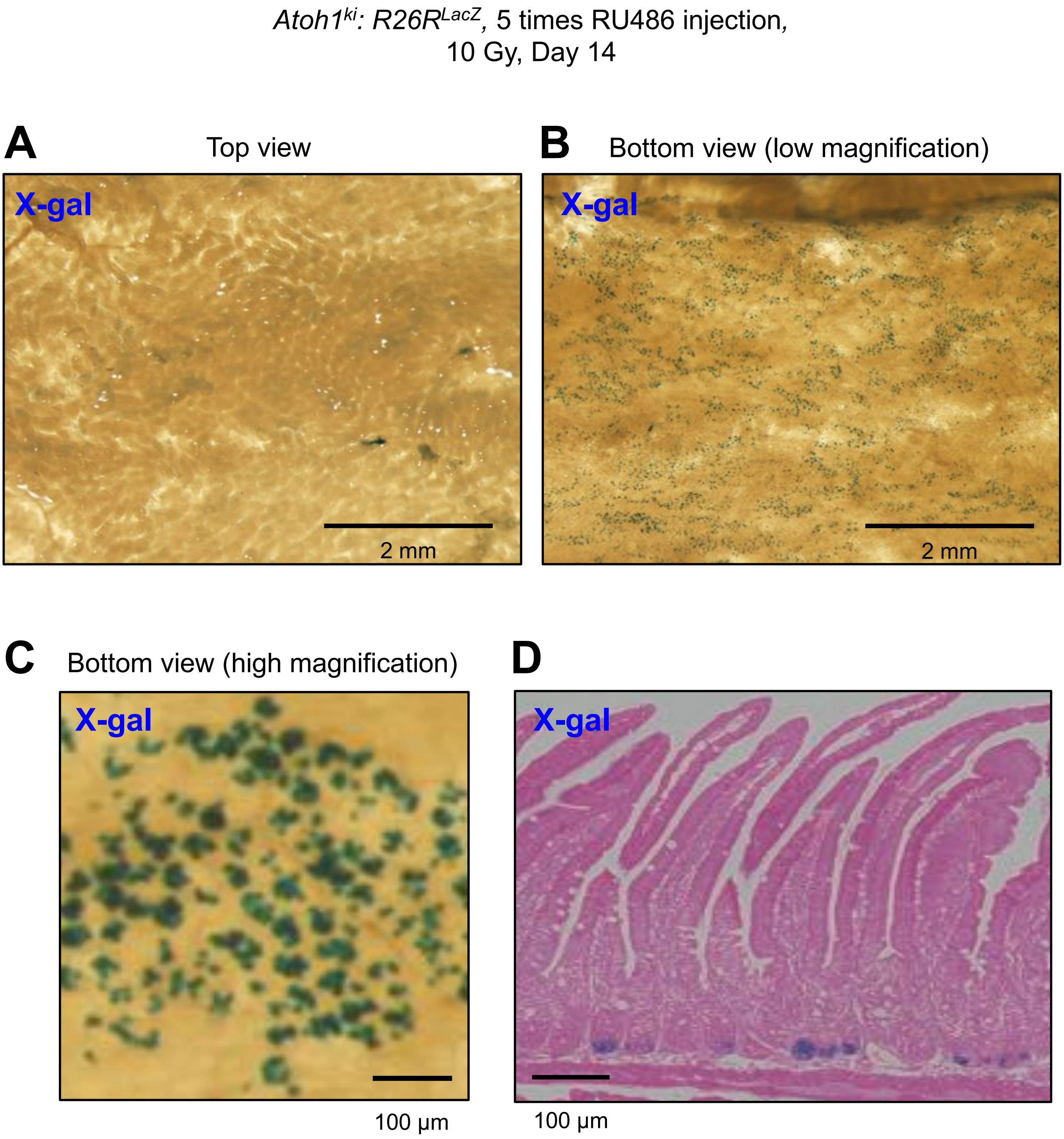

Figure S3. Minor Contribution of Secretory Progenitors to Epithelial Regeneration after Irradiation Damage, Related to Figure 2. Representative macroscopic view (**A**: Top view, **B**: Bottom view at low magnification, **C**: Bottom view at high magnification) and histologic analysis (**D**) of X-gal-stained jejunum 14 days after 10 Gy radiation in *Atoh1^{ki}*: *R26R^{LacZ}* mice that had received five times injections of RU486 as reported previously[19] (n = 3). Note that all X-gal stained cells were Paneth cells, and the remarkable clonal ribbon derived from Atoh1^{+} cells was not found. Scale bars, 2 mm (**A**, **B**), 100 μm (**C**, **D**).

Supplementary Figure S4. Sato et al.

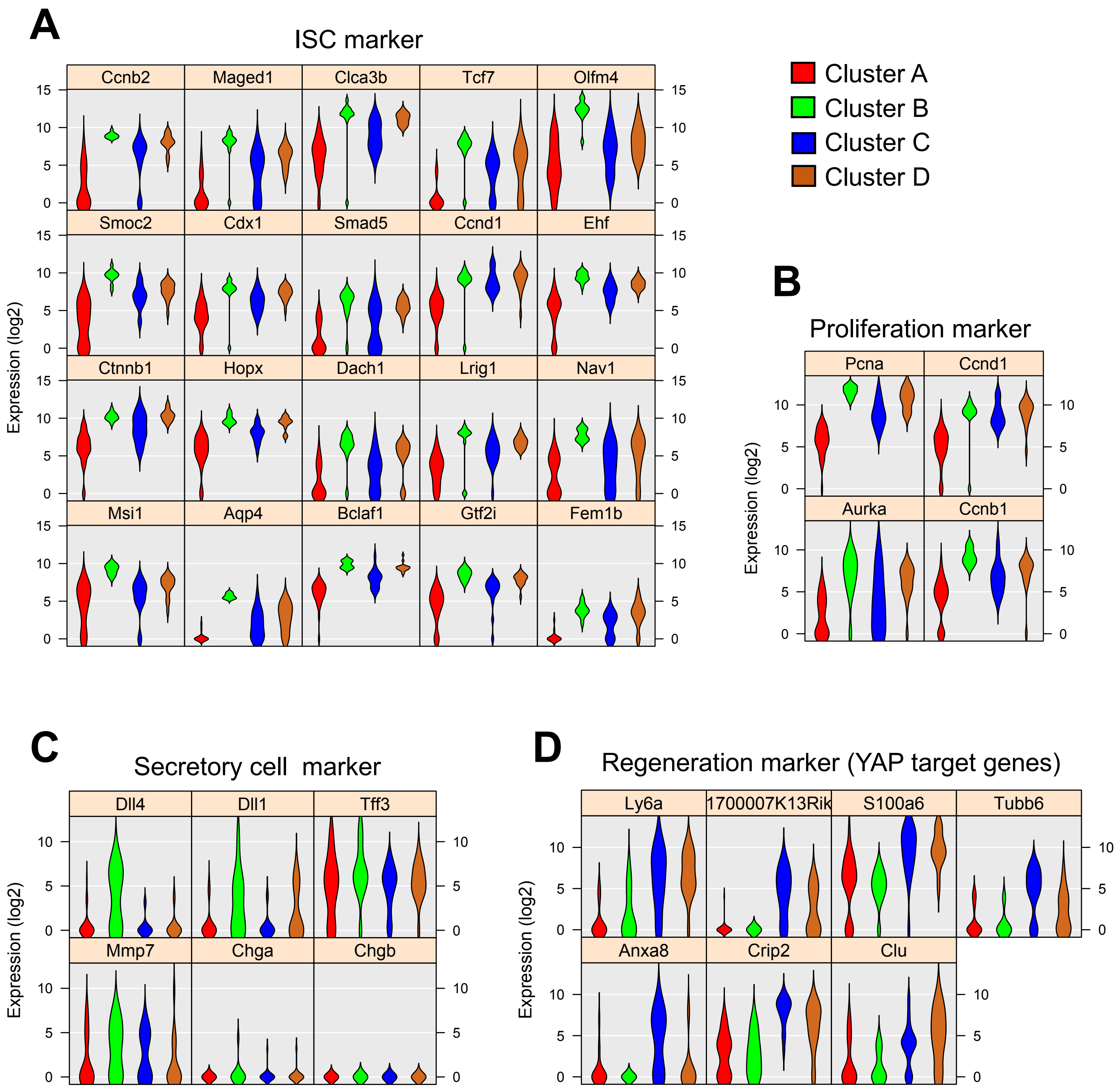

Figure S4. Surviving Lgr5-Derivatives after Irradiation Damage are Heterogeneous, Related to Figure 4. Violin plots of the expression levels of representative genes, such as ISC markers (**A**), Proliferation markers (**B**), Secretory cell markers (**C**), Regeneration markers (YAP targets) (**D**), in each cluster.

Supplementary Figure S5. Sato et al.

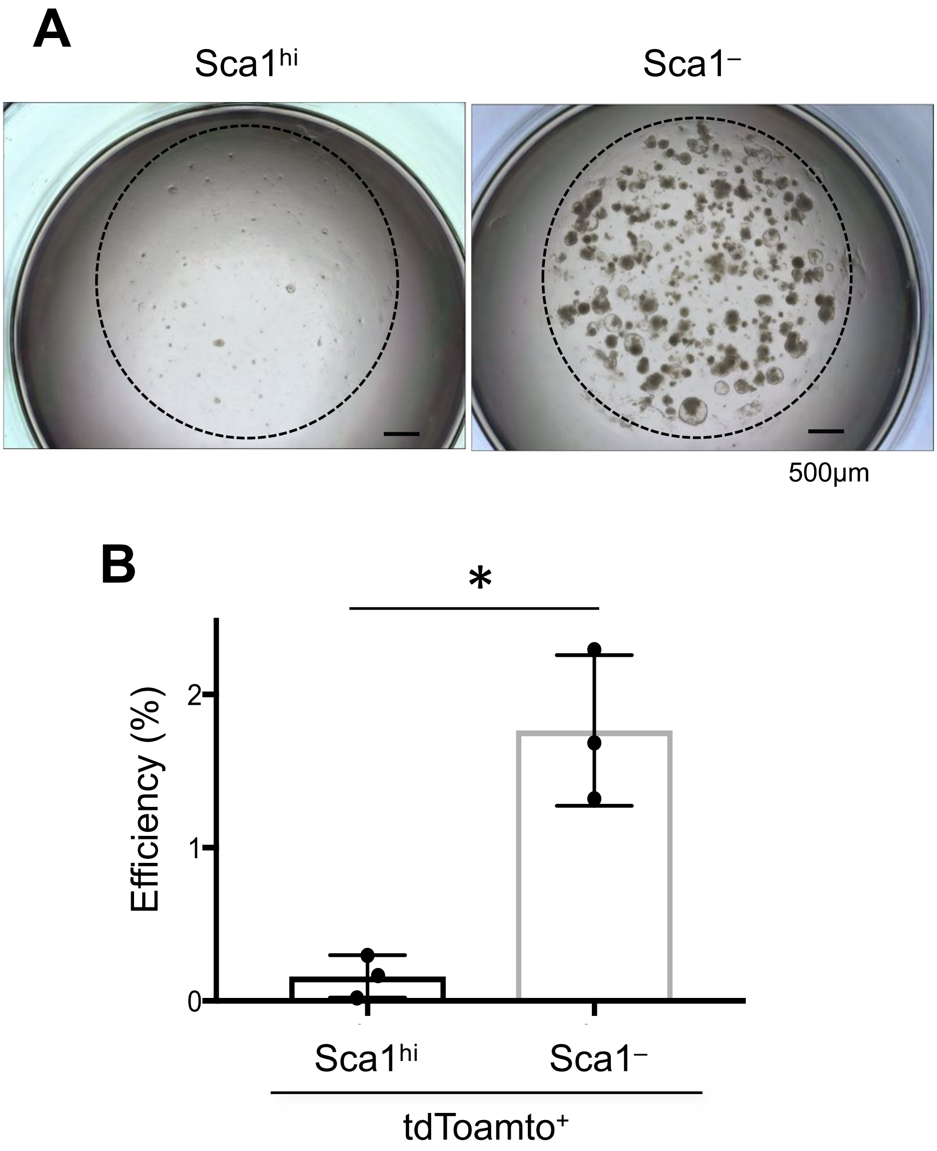

Figure S5. Sca1⁻Lgr5-Derivatives have higher organoid formation potential than Sca1^{hi} cells. Related to Figure 5. Representative images (**A**) and the efficiency (**B**) of organoids generated from either Sca1^{neg} or Sca1^{hi} Lgr5-derivetives prepared from the small intestine of *Lgr5^{ki}*: *R26R^{tdTomato}* mice 3 days after 10 Gy irradiation (n = 3). Data shows mean ± SD of three mice. Each dot represents the data from an individual mouse. Scale bars, 500 µm.*, *p*<0.05 in a Welch's t test.

Supplementary Figure S6. Sato et al.

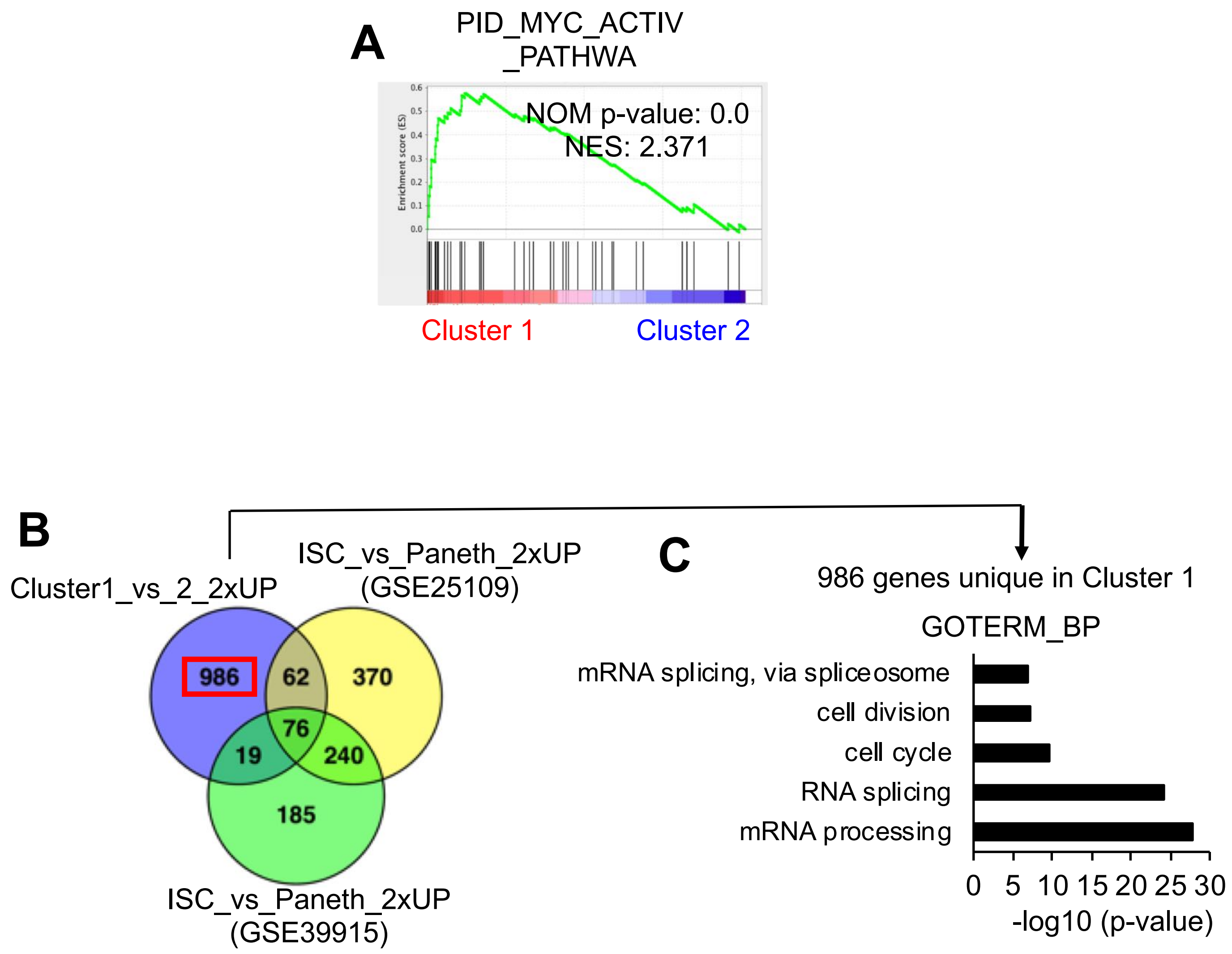

Figure S6. GSEA of proliferation signature in Cluster 1 vs 2 cells, Related to Figure 6. (**A**) PID_MYC_ACTIV_PATHWAY signature in Cluster 1 vs 2 cells. (**B**) Venn diagram showing differentially expressed genes between Cluster 1 overrepresented genes (more than 2-fold higher than Cluster 2) and two different lists of ISC versus Paneth cell signature genes (GSE25109, GSE39915). Cluster 1 cells uniquely expressed 986 genes. (**C**) GO biological processes enriched in the 986 genes identified in **B**.

Supplementary Figure S7. Sato et al.

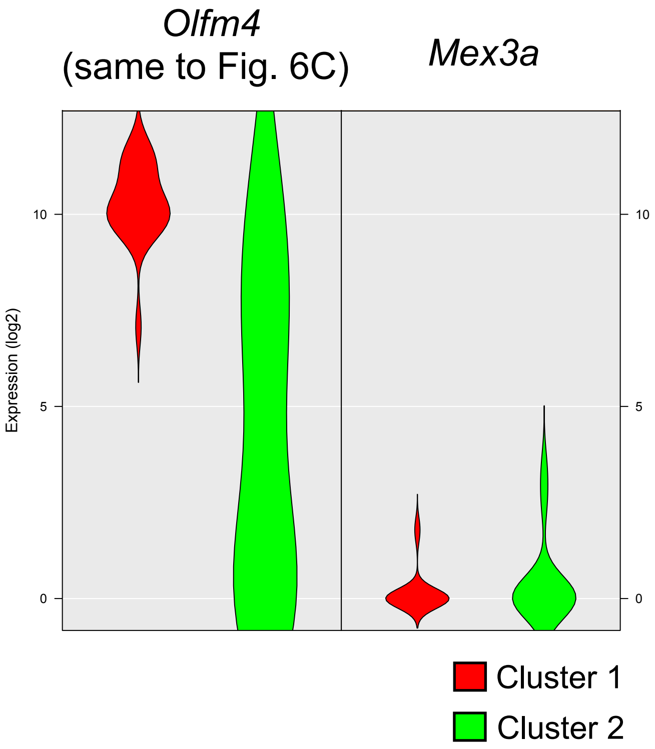

Figure S7. Mex3a expression in Cluster 1 and 2 cells, Related to Figure 6.
Lgr5^{ki}: *R26R*^{tdTomato} mice were injected tamoxifen at 24 hrs before 10 Gy irradiation. Forty-eight hrs later, Sca1^{-}Lgr5-derivatives were isolated and single cell RNA-seq analysis was performed. Violin plot of expression levels of *Olfm4* (same to Fig4C for comparison) and *Mex3a* in each cluster.

Supplementary Figure S8. Sato et al.

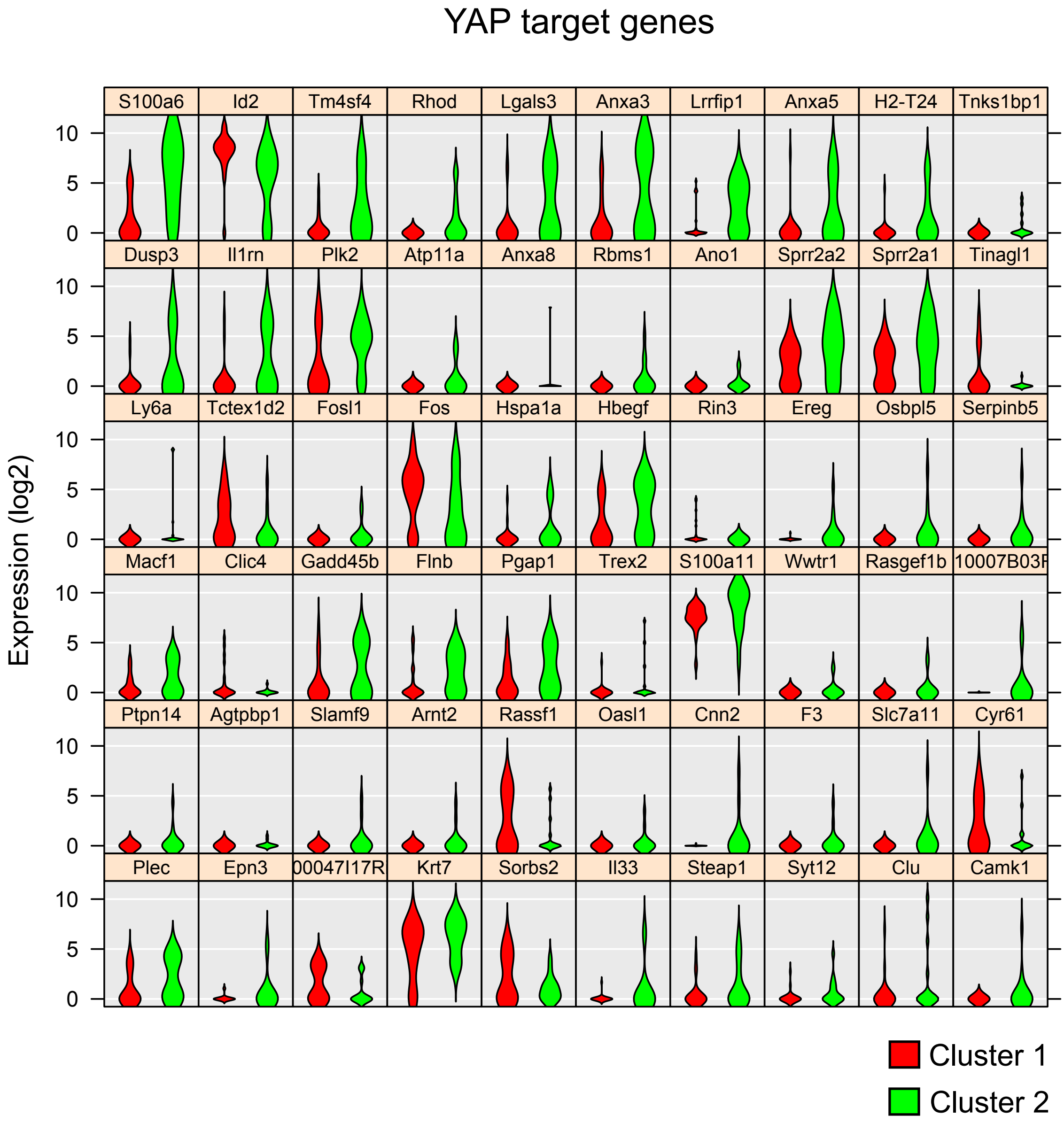

Figure S8. YAP target gene expression in Cluster 1 and 2 cells, Related to Figure 6. *Lgr5*^{ki}: *R26R*^{tdTomato} mice were injected with tamoxifen 24 h before 10 Gy irradiation. Forty-eight hrs later, Sca1^{-}Lgr5-derivatives were isolated and single cell RNA-seq analysis was performed. Violin plot of expression levels of YAP target gens in Cluster 1 and 2 cells are shown.

Supplementary Figure S9. Sato et al.

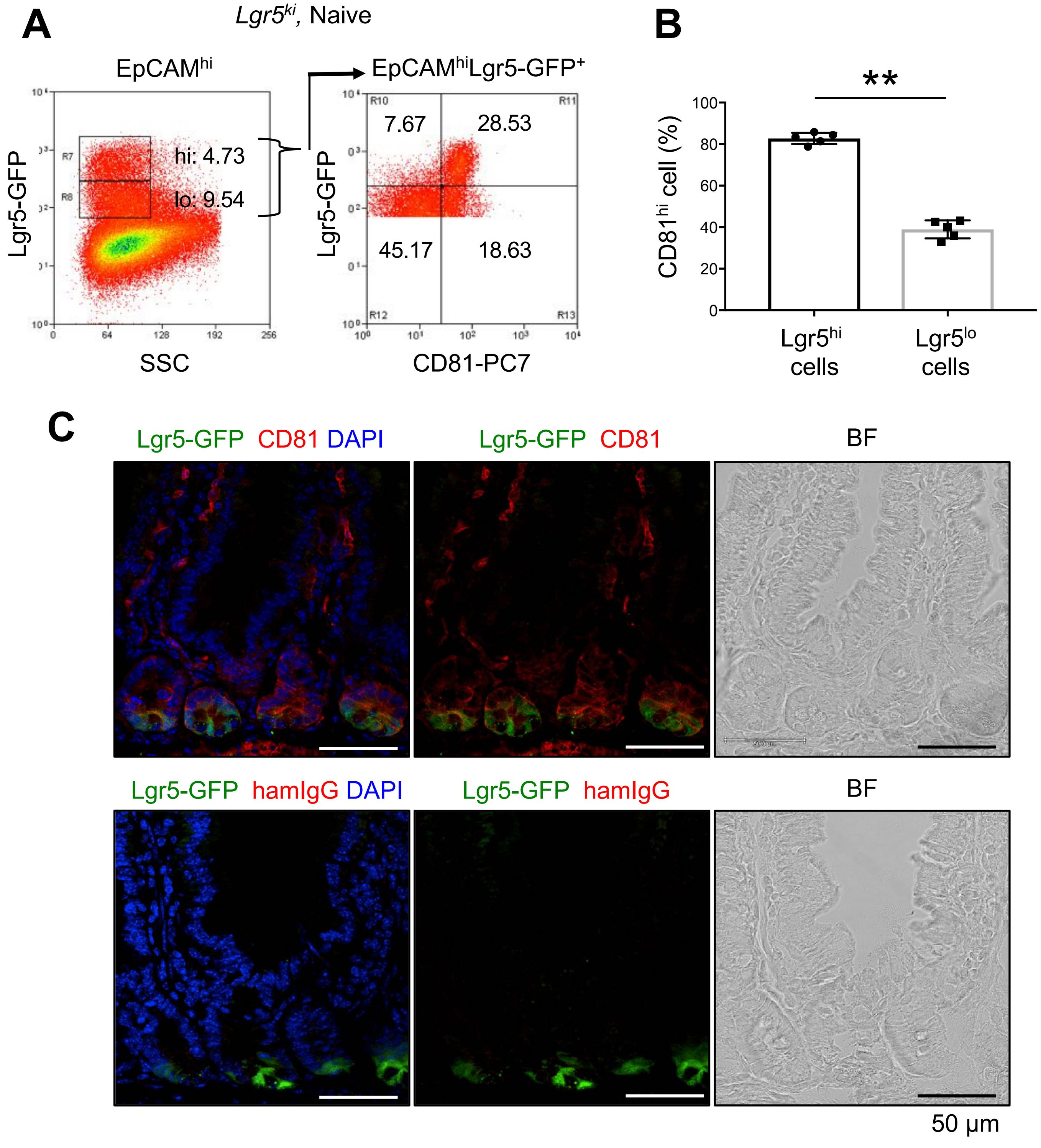

Figure S9. CD81 expression on intestinal epithelial cells in naive *Lgr5^{ki}* mice, Related to Figure 7.

(**A**) Representative FCM profile of crypt epithelial cells of naive *Lgr5^{ki}* mice stained for EpCAM and CD81. CD81 was expressed exclusively on the Lgr5^{hi} ISCs. (n = 5) (**B**) The percentage of CD81^{hi} cells within either Lgr5^{hi} or Lgr5^{lo} cells. Data shows mean ± SD of five mice. Each dot represents the data from an individual mouse.**, p<0.01 in a two-tailed Mann-Whitney test. (**C**) Representative images of the jejunum of naive *Lgr5^{ki}* mice stained for CD81 (upper) or isotype control Ab (lower). Nuclear staining was performed with DAPI. Scale bars, 50 µm. (n = 3)

Supplementary Figure S10. Sato et al.

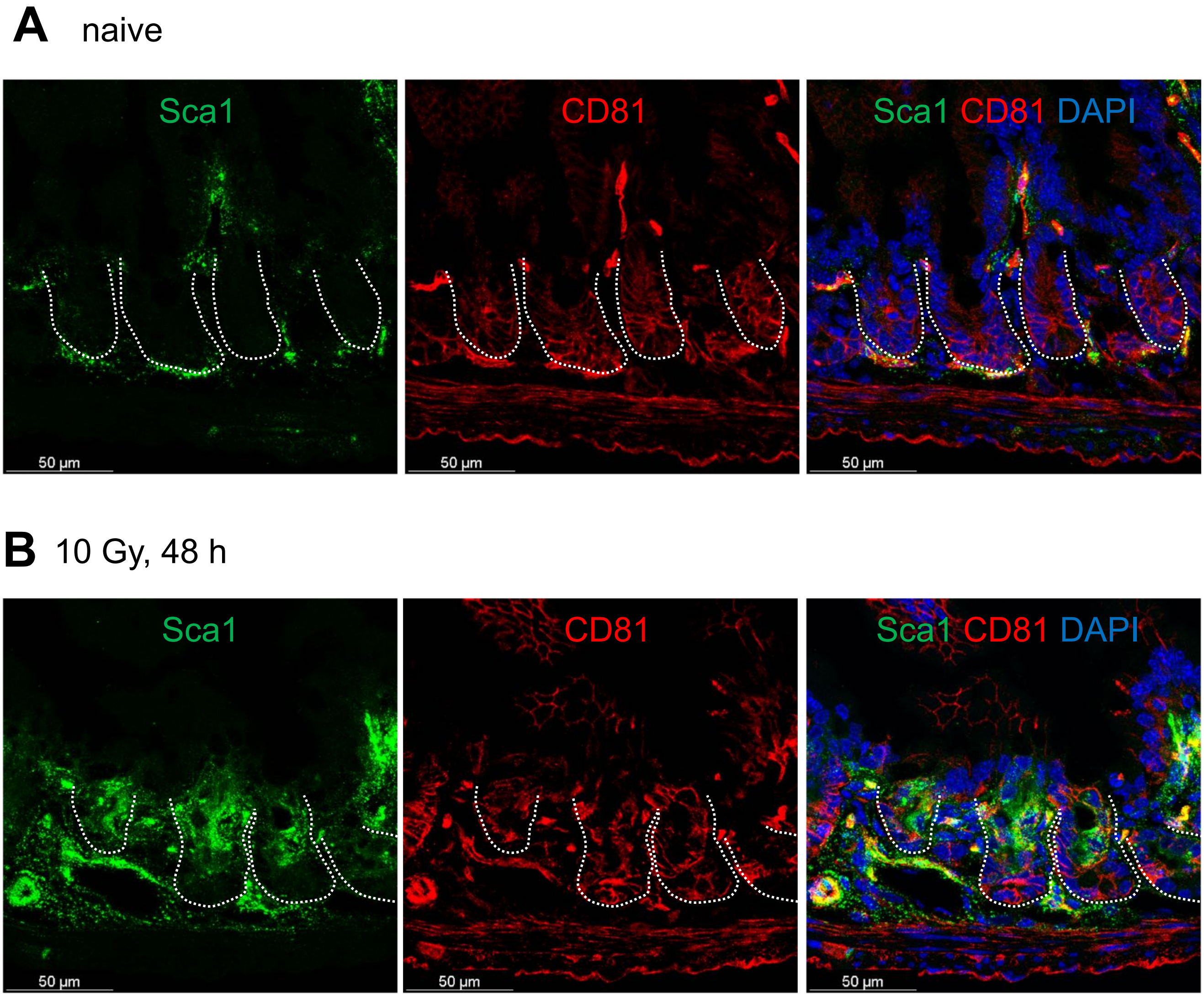

Figure S10. Co-immunostaining of CD81 and Sca1 on intestinal epithelial cells in naïve and in 10 Gy irradiated WT mice, Related to Figure 7. Representative images of the jejunum of naive WT mice (**A**) (n = 3) or 10 Gy irradiated WT mice (**B**) (n = 4) stained for CD81 and Sca1. Nuclear staining was performed with DAPI. Crypts are highlighted by white lines. Scale bars, 50 µm.

Supplementary Table S1. Gene sets for GSEA analysis. Sato et al.

ISC signature	
GSE33949	
The TOP genes of higher expression in Lgr5(hi) cells compared with Lgr5(lo) cells.	
CYP2E1	CD44
LGR5	NOTCH1
TNFRSF19	BHLHB9
FSTL1	RNF43
GKN3	PRSS23
SCN2B	DDO
CES1D	ARHGEF4
PRELP	CLCA1
AFAP1L1	IFITM3
PCDH8	WFDC15B
SP5	EVL
E230029C05RIK	UTRN
RASSF5	ESRRG
SLC14A1	ESAM
ASCL2	CDK6
IL17RD	AQP4
SORBS2	FGFR4
NAV1	SEMA7A
SLCO3A1	CGNL1
FMNL2	WWTR1
HMGCS2	SORCS2
OLFM4	RDH16
ARL4C	RAD51B
CTTNBP2	SLC12A2
AGR3	ZFP618
RGMB	IRS1
ANGPT2	MPP3
AXIN2	TCAF1
HUNK	MECOM
NR2E3	TTC21B
CAR12	CDO1
PTPRO	LECT2
ARID5B	PLA2G4A
DCT	ATG16L2
MSI1	EPHA4
SFRP5	TNS3
SYCN	SMO
SOAT1	CPN1
SMARCD3	PHLDB2
SESN3	H2-EB1
CLCA2	IFFO2
CASP12	KLHL8
EMP2	RNF32
MIA	FZD2
AK7	SMOC2
CDCA7	D930015E06RIK
ACOT1	TGIF2
ZBTB38	PTPRM
PSRC1	ZC3HAV1L

Supplementary Table S1. Gene sets for GSEA analysis. Sato et al. (continue)

YAP signal signature								
GSE66567								
The genes most influenced by YAP signaling in small intestinal epithelium								
SERPINB9B	GCNT1	MX2	SLC6A14	2010002M12RIK	CAR2	LMO7	RTP4	SUOX
LY6C1	TCEAL7	TEAD4	NRIP3	SLC25A48	VGLL3	PIM1	1700012L04RIK	PDGFB
GGTA1	ARHGAP23	FOSL1	CISH	BEX1	KLF6	TULP2	CWH43	BAIAP2
SYT8	RASSF8	RNF208	LATS2	CD55	S100A11	PTRH1	DENND2C	MCAM
PSCA	CLU	KRT7	CRIP2	ADAMTS15	S100A6	SH3TC1	PWWP2B	KRT9
CTGF	DUSP14	LAMC2	ILDR1	CDKN1C	ID1	ARNT2	CNKSR1	NCEH1
CYP1A1	SPRR2A2	CLCA4A	SORBS2	PEAR1	ISG20	GADD45B	TCTE2	OSBPL3
KRT80	SPRR2A1	FAM129A	MRC1	DUSP9	SUSD2	MAPK11	GLT28D2	KRT17
MSLN	ANO1	ISX	ZMYND15	SLC7A11	CAPN5	FAM160A1	CNEP1R1	
TM4SF1	ANXA3	APOL9A	CAV2	SERTAD4	GML	MACF1	METTL7B	
ANKRD35	EPHA2	CAPN2	DUSP10	SH3TC2	ABHD2	ACOT9	GLIPR1	
ZFP185	VNN1	MYOF	HSPA1A	SEMA3C	SLC5A6	GJB3	TJP1	
IL33	ATP11A	ISG15	LAMB3	WWC1	PPL	PHLDA1	1700047I17RIK2	
WNT7A	STBD1	WWTR1	DLK2	MYCBPAP	PRKCDBP	LRRFIP1	GRB10	
PLAU	PTRF	DDX60	MYO1C	F3	KLF4	PHLDA2	EGFR	
EHD2	SPRR2B	ANXA1	MMP23	TRPM6	TM4SF4	SH3BP5	DHX58	
CALCB	RRAD	STX11	PDLIM2	ADAMTSL4	CLIC4	PLCD3	NEU1	
HPCAL4	ZFP37	HSPA1B	FLNA	EMP2	STON1	PAQR8	CRLF3	
AKAP2	IFIT3	GLIS2	ANXA8	PPP1R15A	PMAIP1	SLC44A2	TRIB3	
FGD3	SYT12	HDAC7	LAMB2	LYNX1	TMEM237	CHAC1	HPSE	
SYDE1	XAF1	EPN3	EMP1	PRSS23	DERL3	PODXL	STC2	
NID1	ARHGAP40	PTPN14	MRAS	LAMA3	PRRG4	ASAP1	ANGPTL6	
MNDAL	BMP2	APOL9B	PRL2C3	RHOD	PPP1R9A	EDN3	FAM43A	
LOR	SERPINB9	NT5E	BCAM	WTIP	S1PR2	LITAF	ARID3A	
LY6C2	PHLDB2	RNF39	IL1RN	PARD6B	ARL4C	PTPRT	GDF15	
P2RX2	2010109I03RIK	2310007B03RIK	TIMP2	CIDEC	JAG2	SERPINB5	DUSP8	
BTN1A1	PCOLCE	S100A14	FAM189A2	HBEGF	1700016K19RIK	ANXA5	TGFBR2	
YAP1	GNGT2	LGALS3	DOK2	SGK2	ID2	MAFF	PAM	
ILDR2	AHNAK	PLK2	KIFC3	HIST4H4	GM7008	HERC6	FAM83H	
WWC2	FABP3	PAK6	NPNT	BASP1	RRAS2	TCTEX1D2	OTUD7B	
EDN1	RSAD2	KLRG2	SMTNL2	TPM2	ARHGEF17	FLNB	PGAP1	
CLIC3	USP18	SPON1	CLCF1	CAMK1	PRND	RHOF	PVR	
CRYAB	DPCR1	SLC5A5	TNFRSF12A	ACOX2	CGNL1	TSC22D2	IFNLR1	
SYT16	LAMA5	THBS1	KLHL13	BMP8B	1700019L03RIK	IFRD1	S100A16	
SLC25A43	PMP22	RIN3	ZBP1	E130012A19RIK	BICD1	TRIM15	RAPH1	
PLAT	CXCL10	TGM1	TREX2	ADM2	MDFI	SSH1	OTUD1	
AMOTL2	HIST1H3C	CHRNB1	CXCL16	RASGEF1B	AREG	EREG	PLEKHG3	
ARHGAP22	RBPMS	PCSK6	ZFP57	TLN2	GRASP	RUSC2	TSPAN4	
FGF15	GPRC5A	CNN2	SLAMF9	ARC	PTPN21	RASSF1	KRT14	
ADAM8	1700056E22RIK	HIST1H1E	S100A4	OASL2	FOS	STEAP1	KCTD10	
LY6A	OXCT2B	DNAH2	PPP1R2	PDZK1IP1	PALLD	TRIP6	KCTD11	
UNC13D	AMOTL1	OMP	GM14446	ITGB6	KRT18	SEZ6L2	PCYT1A	
SLC12A4	RND1	AKAP5	RIN1	VANGL1	SAMD9L	ACOT10	IL3RA	
CDH16	STK32C	OAS3	ADM	DUSP3	OSBPL5	KRT23	H2-T24	
WFDC2	TINAGL1	IFIT1	SLC9A4	CCDC120	STEAP2	TNKS1BP1	TBC1D2	
GJB4	A430105I19RIK	NBL1	ARHGAP29	MBOAT1	RBMS1	HIST1H2BJ	MYO1H	
CYR61	SLC7A3	ABCC5	CSRNP1	CCDC68	CCL20	PPARG	CAPN13	
MAP6	JDP2	ATF3	NR4A1	TUFT1	CLDN4	OASL1	KRT16	
PLAUR	DUSP1	FBXO24	EMP3	ECEL1	AKR1B8	PLEC	FLOT2	
I830012O16RIK	IER3	SLC35E4	LDHD	FAM46B	AGTPBP1	SMAD7	TAGLN2	
SYNPO2L	LHFPL2	HMOX1	SPRR1A	ID3	IRGM2	ARID5A	RAPGEF2	

Supplementary Table S2. Primers for qRT-PCR. Sato et al.

Target	Forward (5'-3')	Reverse (5'-3')
Hprt	GACCTCTCGAAGTGTTGGATAC	CTTGCGCTCATCTTAGGCT
Olfm4	GCCACTTTCCAATTTCAC	GAGCCTCTTCTCATACAC
Lgr5	GACAATGCTCTCACAGAC	GGAGTGGATTCTATTATTATGG
Fstl1	CACGGCGAGGAGGAACCTA	TCTTGCCATTACTGCCACACA
Atoh1	GAGTGGGCTGAGGTAAAAGAGT	GGTCGGTGCTATCCAGGAG
Spdef	TTGGATGAGCACTCGCTAGA	AGCCGGTACTGGTGTTCTGT
Lyz1	GGAATGGATGGCTACCGTGG	CATGCCACCCATGCTCGAAT
Defa6	CCTTCCAGGTCCAGGCTGAT	TGAGAAGTGGTCATCAGGCAC
Muc2	GTCCGAAGTGTTACCCTGGA	CCAGGAGTGGAGAAGGTCAG
Mki67	ATCATTGACCGCTCCTTTAGGT	GCTCGCCTTGATGGTTCCT

Supplementary Table S3. Primers for single cell qRT-PCR. Sato et al.

Target	Forward (5'-3')	Reverse (5'-3')
1700007K13Rik	AGTAACCAAACCTACGGAAGCA	GCTGCGTGTTGTCTGGAAAA
Alpi	TCCTAAAGGGGCAGTTGGAA	ACCTGTCTGTCCACGTTGTA
Anxa1	GTGACATTGAGAAGTGCCTCAC	TCGTACAGCTTCTCGGCAAA
Anxa8	AAGATGTACGGCAAGACTCTCA	TTCAGCAGGGCAGTCTTGTA
Aqp4	GGCATCCTCTACCTGGTCAC	CCAGCGGTGAGGTTTCCA
Atm	GCTTAGAAAACCCTGCAGTCA	GCCATCGTAACTTCCAGCAA
Aurka	CCTGTGTCCCGGCTCAATAA	AAGGATGCCTGCTCCTTTTCA
Bclaf1	GCAAAAGAGCCCCGAGATAC	AACCTCTCTCCTCTCCTGCTA
Brca2	ACACCACCAACCCTTAGTTCC	GAGAGTCAGCAGGCGTTACA
Car2	TCAACAACGGCCACTCCTTTA	ACTGAGGGGTCCTCCTTTCA
Cbfb	CCAGAGGAGCAAGTTCGAGAA	CCCTGAAGCCCGTGTACTTA
Ccnb1	GCTGCTTCAGGAGACCATGTA	TAGCATCTTCTTGGGCACACA
Ccnb2	GCCTCTTGCCTGTCTCAGAA	CACTCTCCATGTAGCCTGTGTA
Clca1	ACAACCACTAAGGTGGCCTA	GAGCTCGCTTGAATGCTGTA
Clca3b	GGTCTGCTTGGTGCTGGATA	AGCTCTGCTGCTTGGTTCA
Clu	AACTCCACAGGATGCCTGAA	GGGCAGGATTGTTGGTTGAA
Creb3l4	CCCTCCGATTCGCATAGACA	GAACAGATGATGTTGGCAGGAC
Crip2	TGTCCCAAGTGTGACAAGACC	AGACAGAACTTGTGCCAGTCC
Ctnnb1	CATTGGTGCCCAGGGAGAA	GCCGTATCCACCAGAGTGAAA
Cyp2e1	ATGGGCTCCTGATTCTCATGAA	TTGGCCCAATAACCCTGTCA
Dach1	TGACATGGGGCATGAGTCAAA	TCTTGCGGTTGGTGTGGAA
Defa3	CCCAGAAGGCTCTTCTCTTCA	CTTTCTGCAGGTCCCATTCA
Eda2r	GCTGTGTCACCTGCCAAC	TGTGCATCTCCACCTTCTCC
Ehf	AGCTGCTCTACAGCAACCTA	CATTGTGTGCGGACTGGAAA
Fem1b	TGCGCTTGCTGTTAGAACAC	AGCAGTGGCACCGTCAA
Foxa3	TGGCCGAGTGGAGCTACTA	GGCACAGGATTCACTGGAGAA
Foxj2	GTCCTTCCGCAACCTCTACAA	TGTCTCCCAGGAGTGAGGAAA
Gja1	TCAGCCTCCAAGGAGTTCCA	ACCTTGTCCAGCAGCTTCC
Gtf2i	GGCCCCATCAAAGTGAAAAC	TGACTCCTCCTTCACTGTCA
Il33	GGTCCCGCCTTGCAAAATA	AGAACGGAGTCTCATGCAGTA
Kcnq1	TCATCAGGCGCATGCAGTA	ATGACATCTCGCACGTCGTA
Kit	GTGCCAACCAAGACAGACAA	TTCCATGATGGCAGGAGTCA
Lbh	AGGTGATGATGAACGCTCCA	AAAGTCTGATGGGTCCGGAAA
Ly6a	GTTACTCAGGAGGCAGCAGTTA	AGGGCAGATGGGTAAGCAAA
Maged1	GCTCCTACCATGAGACTAGCA	GAACTGTGCAGTCCAGTCAC
Mxd4	GCGACTTCGCAAGGAAGAAA	TGCTTTTCTAGTTCGTTGTGTGAA
Mycl	GGCGAGCCCAAGACTCA	CCAGAGATCGCCTCTTCTCC
Nav1	GGAGCGTTAAATGCCTCAGAA	TGTTGAGGCTGGAGATGCTA
Nkx2-2	CCAGCCTCATCCGTCTCA	CGGGCACGTTTCATCTTGTA
Nr2e3	GGCTGTCAAATGGGCCAAAA	TTCCATGCCTCTTCCAGCAA
Pou2f3	TTCGCCAAGACCTTCAAGCA	GTCATTGCCATACAGCTTTCCC
Prox1	GCCCTCAACATGCACTACAAC	CGTGATCTGCGCAACTTCC
S100a6	AAGCACACCCTGAGCAAGAA	AGCCTTGCAATTTCAGCATCC
Smad5	CCCAGCCTATGGATACAAGCA	CTCATAGGCGACAGGCTGAA
Smoc2	CCGGGACCTGTACAAGAACA	CATCCAGGACACTTGTCAGAAAC
Spdef	GCCTGCAAGCTTCTGAACA	AAGCCACTTCTGCACGTTAC
St18	TCATGGCTGCCAACTCTCAA	GGAGGCATAGTTTCCAGTCACA
Tacstd2	TGACGGTCTGCGACACAAA	CGTGGAGCAGTCGACCAATA
Tcf7	AAGAGGCGGTCAAGGGAAAA	GGCCTTCTCCGGGTAAGTAC
Tff3	AAATGTGCCCTGGTGCTTCA	GGAGCCTGGACAGCTTCAAA
Trim24	ACCACACAGTCAGGCAGAA	ACAAAACACTGGTCGCTGAC
Tubb6	AGGGAGATTGTGCACATCCA	GTGCTCATCACTGATCACTTCC
Chga	CCCGAAGTGACTTTGAGGAA	ATGGCTGACAGGCTCTCTA
Lyz2	AAGGAATGGAATGGCTGGCTA	ATTGCTCTCGTGCTGAGCTA
Mmp7	CCCGGTACTGTGATGTACCC	AATGGAGGACCCAGTGAGTG
Dll1	TGAGCCAGTCTTTCCTTGAA	AGACCCGAAGTGCCTTTGTA
Dll4	GAGAAGGTGCCACTTCGGTTA	TAGAGTCCCTGGGAGAGCAAA

Supplementary Table S3. Sato et al. (Continue)

Target	Forward (5'-3')	Reverse (5'-3')
Muc2	GTCCGAAGTGTTACCCTGGA	CCAGGAGTGGAGAAGGTCAG
Atoh1	TCGTATCTCTGCCTCTGGTCT	TTTGTGAGTGAGCGCAACAC
Olfm4	AGGCACTTCTTGGGCAGAA	TGTCCACAGACCCAGTGAAAT
Lgr5	ACGCCTTTGGAAACCTCTCC	CAGGCTGTGGAGTCCATCAAA
Msi1	GCCATGCTGATGTTCGACAA	CTACGATGTCCTCGCTCTCAA
Axin2	GATCCACGGAAACAGCTGAA	AGCCGGAACCTACGTGATAA
Cd133	ATCGGGGAAACGAAGAAGTT	ACAGCCGGAAGTAAGAGCAC
Sox9	AGTACCCGCATCTGCACAA	GTCTCTTCTCGCTCTCGTTCA
Notch1	GGACGGCGTGAATACCTACA	GACATTCGTCCACATCCTCTGTA
Ascl2	CAGGAGCTGCTTGACTTTTCC	GTAGGTCCACCAGGAGTCAC
Bmi1	GCAGCAATGACTGTGATGCA	CAATGTCCATTAGCGTGTAGTAATCC
Hopx	GTCTCACGGAGGAGCAGAC	GTCCGTAACAGATCTGCATTCC
Lrig1	GCTCCACATACTCCCACAAA	GATGGCAATGTCATGGGGTA
Ccnd1	CTCTCCTGCTACCGCACAA	CTTGACTCCAGAAGGGCTTCA
Pcna	TGGACTTAGATGTGGAGCAACTT	TTCACCCGACGGCATCTTTA
Gapdh	AGAGACGGCCGCATCTTC	TTCACACCGACCTTCACCATT
Cdx1	GCTAACCTGGGGCTCACA	GTGTGGGAGTGCCATCCA
